# An NTP-driven mechanism for the nucleotide addition cycle of *Escherichia coli* RNA polymerase during transcription

**DOI:** 10.1371/journal.pone.0273746

**Published:** 2022-10-25

**Authors:** Ronald S. Johnson, Mark Strausbauch, Christopher McCloud

**Affiliations:** Department of Biochemistry and Molecular Biology, Brody School of Medicine, East Carolina University, Greenville, North Carolina, United States of America; Consejo Superior de Investigaciones Cientificas, SPAIN

## Abstract

The elementary steps of transcription as catalyzed by *E*. *coli* RNA polymerase during one and two rounds of the nucleotide addition cycle (NAC) were resolved in rapid kinetic studies. Modelling of stopped-flow kinetic data of pyrophosphate release in a coupled enzyme assay during one round of the NAC indicates that the rate of pyrophosphate release is significantly less than that for nucleotide incorporation. Upon modelling of the stopped-flow kinetic data for pyrophosphate release during two rounds of the NAC, it was observed that the presence of the next nucleotide for incorporation increases the rate of release of the first pyrophosphate equivalent; incorrect nucleotides for incorporation had no effect on the rate of pyrophosphate release. Although the next nucleotide for incorporation increases the rate of pyrophosphate release, it is still significantly less than the rate of incorporation of the first nucleotide. The results from the stopped-flow kinetic studies were confirmed by using quench-flow followed by thin-layer chromatography (QF-TLC) with only the first nucleotide for incorporation labeled on the gamma phosphate with ^32^P to monitor pyrophosphate release. Collectively, the results are consistent with an NTP-driven model for the NAC in which the binding of the next cognate nucleotide for incorporation causes a synergistic conformational change in the enzyme that triggers the more rapid release of pyrophosphate, translocation of the enzyme along the DNA template strand and nucleotide incorporation.

## Introduction

The nucleotide addition cycle (NAC) of RNA polymerase during transcription is a multistep process involving translocation of the enzyme along the DNA template, entry of nucleotides into the active (*i + 1*) site, chemistry leading to nucleotide incorporation and the release of pyrophosphate. X-ray crystallographic and biochemical studies indicate that these steps are accompanied by structural alterations in mobile domains of the enzyme such as the bridge helix and the trigger loop [1–10]. There are three major models that have been proposed for the NAC. In the classical Brownian-ratchet model [11–18], RNA polymerase is thought to oscillate between the pre- and post-translocated states. This oscillation allows nucleotides to enter the active site when RNA polymerase is in the post-translocated state and the secondary channel leading to the active site is open. The nucleotide bound at the active site acts as a pawl to trap the enzyme in the post-translocated state thereby allowing it to undergo incorporation. In the allosteric model [19, 20], the next nucleotide for incorporation is thought to interact with an allosteric site that converts the elongation complex to an activated state. In the power stroke mechanism, it has been postulated that the translocation of RNA polymerase from the pre- to the post-translocation state is driven by some chemical process. There are several variations of this mechanism. In the case of T7 RNA polymerase, it has been postulated that the release of pyrophosphate after nucleotide incorporation generates a change in protein conformation that drives strand separation and translocation [21]. In the case of prokaryotic and eukaryotic RNA polymerases, it has been postulated that the next cognate nucleotide for incorporation binds to the pre-translocated state of the elongation complex and triggers translocation with subsequent nucleotide incorporation by eliciting a conformational change in the protein [22–25].

The prevailing view is that the NAC in prokaryotic and eukaryotic RNA polymerases proceeds by a Brownian-ratchet model in part because the other models require the binding of nucleotides to downstream DNA at *i + 2* or greater. This would necessitate nucleotides accessing the DNA through the primary channel which contains the downstream double stranded DNA along with the downstream single stranded DNA template strand. However, molecular dynamic (MD) simulations with eukaryotic RNA polymerase II in the absence of the TFIIF transcription factor indicate that conformational and electrostatic constraints prevent nucleotides from accessing the downstream DNA through the primary channel [26]. In contrast, MD simulations indicate that nucleotides can readily access the active (*i + 1)* site through the secondary channel [27, 28]. When accelerated molecular dynamic (aMD) simulations were conducted on eukaryotic RNA polymerase II in the presence of the TFIIF transcription factor, four subchannels collectively referred to as the tertiary channels (3A/B/C/D) were identified [29, 30]. These subchannels begin at different sites on the surface of the enzyme and then merge into a cavity referred to as the channel 3 pocket (CH3P) that contains the *i + 2* site. A reinvestigation of the crystal structures of *E*. *coli* initiation complexes with complete DNA bubbles (PDB#4YLN, 4YLO and 4YLP) [31] resulted in the identification of channels similar to the RNAPII subchannels 3A/B that lead to the *i +2* site on the template DNA [30]. Identification of these channels provides support for mechanisms of the NAC in which the cognate NTP for incorporation interacts with downstream DNA.

The results from rapid kinetic studies of one and two rounds of the NAC as catalyzed by RNA polymerase in this study are consistent with an NTP-driven mechanism during transcription. A key result in this study supporting an NTP-driven mechanism is the demonstration that the next nucleotide for incorporation increases the rate of release of the pyrophosphate generated by the previously incorporated nucleotide. This is possible if the next nucleotide for incorporation binds to the enzyme at the *i +2* site in the CH3 pocket prior to pyrophosphate release. The interaction of the nucleotide with the *i +2* site in the CH3 pocket could then elicit a synergistic conformational change that leads to the more rapid release of pyrophosphate from the active site, triggers translocation of the RNA core polymerase from the pre- to the post-translocated state and entry of the nucleotide into the active site for incorporation.

## Results

### Pyrophosphate release is slow relative to nucleotide incorporation during one round of the NAC

**Fig 1:**
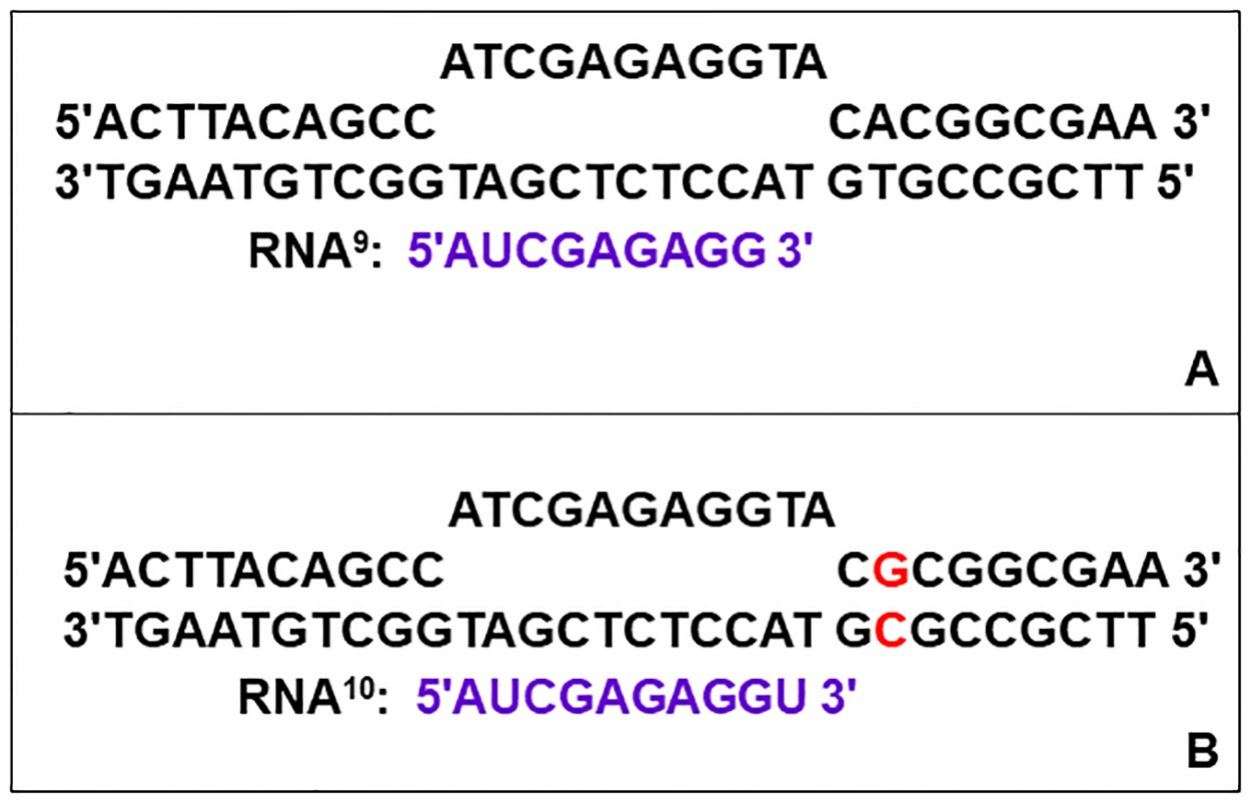
Nucleic acid scaffolds used in association with *E*. *coli* core RNA polymerase to produce well-defined elongation complexes. Each scaffold contained an RNA primer annealed to a 30 nt DNA template strand that is associated with a 30 nt fully complementary non-template strand. (**A**) The scaffold containing the 9mer RNA was used in the stopped-flow kinetic studies. (**B**) The scaffold containing the 10mer RNA was used in the quenched flow-thin layer chromatography (QF-TLC) studies. The base pair given in red indicates the alteration in the DNA sequence that was made to limit the incorporation of nucleotides to only AMP followed by CMP.

The elongation complexes (EC) used in this study are given in Fig 1. For the stopped-flow kinetic studies used to monitor pyrophosphate release during one round of the NAC, the EC contained *E*. *coli* core RNA polymerase in a complex with the nucleic acid scaffold given in Fig 1A containing the 9mer RNA primer. In the presence of UTP, UMP is added to the 3′ end of the primer and pyrophosphate is generated.

**Fig 2:**
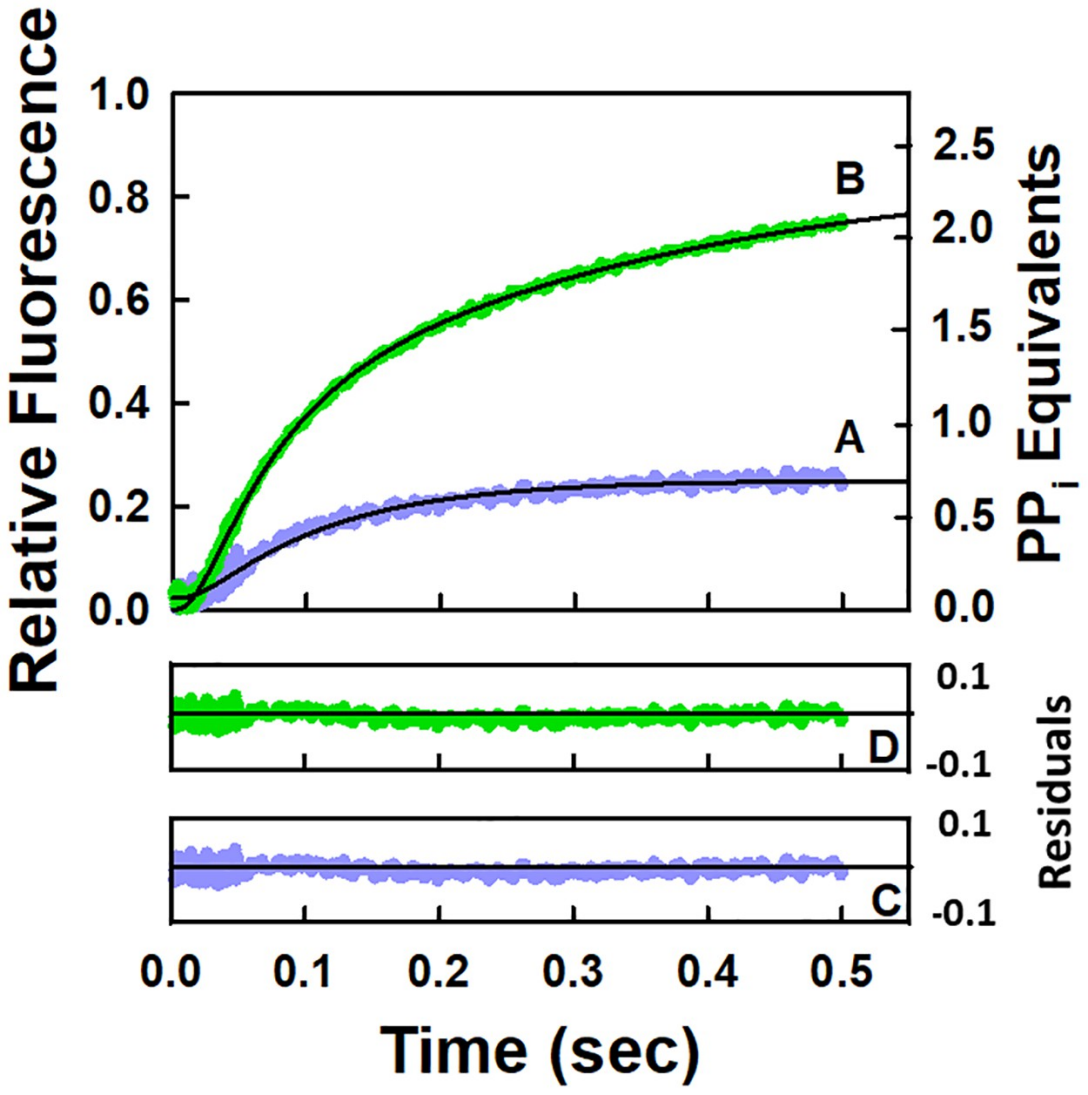
Stopped-flow kinetic results for pyrophosphate release during one (A) and two rounds (B) of the NAC. The reactions were monitored by using a coupled enzyme assay as outlined in the experimental section. In each case, a control in the absence of the EC corresponding to the background was subtracted from the reaction in the presence of the EC and nucleotide(s). The concentration of the EC after mixing was 0.2 μM; 25°C in each case. (**A**) UTP final concentration after mixing was 50 μM; the pyrophosphate release curve corresponds to the average of three runs; and the solid line through the data points corresponds to the fit to Scheme 1. (**B**) UTP and ATP concentrations after mixing were both 50 μM; the pyrophosphate release curve corresponds to the average of two runs; and the solid line through the data points corresponds to the fit to Scheme 4. **(C)** Residual plot for the fit of the data from a single round of the NAC. **(D)** Residual plot for the fit of the data from two rounds of the NAC.

The time dependent release of pyrophosphate for a single round of the NAC is given in Fig 2A. The data were fitted to the model in Scheme 1 by using KinTek Explorer [32, 33] which is used for fitting all the data in this study. The constraints (upper and lower bounds) for the value of each parameter that was allowed to float in the fitting routine were determined by analysis of the FitSpace [33] which is a subroutine of KinTek Explorer. How well the values of the parameters are constrained is a measure of the uncertainties in their values. Scheme 1 as well as all the other schemes corresponds to the minimal model required to identify the major rate limiting steps in the NAC. Studies indicate that RNA polymerase favors the pre-translocated over the post-translocated one [12, 18, 34–37]. As a result, all the schemes were formulated with the elongation complexes initially in the pre-translocated state except for Scheme 2. Because X-ray crystallographic studies indicate that the trigger loop in the β′ subunit of *E*. *coli* RNA polymerase undergoes a conformational change upon NTP binding that positions the NTP for incorporation and traps it at the active site [1–5, 12], this step is formulated as irreversible. The other steps are also formulated as irreversible because nucleotide incorporation as well as pyrophosphate release occur on the order of milliseconds whereas the reverse reaction (pyrophosphorolysis [6, 7, 37, 38]) occurs on the order of minutes. All the schemes in this study are formulated in the same manner. The forward rate constants for all nucleotide binding steps were set equal to a diffusion limited rate constant of 100 μM^-1^ s^-1^ in accord with the transient-state kinetic studies of the NAC for eukaryotic RNA polymerase I [39]. The reported value of the rate constant *(k*_*2*_*)* for UMP incorporation from quench-flow studies is 58 s^-1^ for this EC [40]. Using this as a starting point, the value of *k*_*2*_ was varied to optimize the fit for the stopped-flow data according to the model in Scheme 1. The values of the various rate constants are listed in Table 1. The residuals from fitting the data to the model in Scheme 1 are given in Fig 2C. The first of two criteria that must be satisfied in determining the goodness of a fit to a model is that the residuals must not demonstrate any dramatic and systematic deviations between the data points and the values predicted by the model. The second of the two criteria that must be satisfied is that the fitted values of the different parameters must be well-constrained. In this case, the goodness of the fit based on the residuals (Fig 2C) and the well-constrained values of the fitted parameters (Table 1) support the four-step model in Scheme 1 in which nucleotide binding occurs prior to translocation. The value of the rate constant for pyrophosphate release is approximately seven times less than the value of the rate constant for UMP incorporation during one round of the NAC. The value of the rate constant for translocation is comparable to the ones reported previously (*i*.*e*., 59–96 s^-1^) [41].

**Table 1:** Values and limits of rate constants for *E*. *coli* RNA core polymerase during one round of the NAC based on the model given in Scheme 1 for UTP binding to the pre-translocated state as monitored in stopped flow kinetic studies.

Parameter	Value	Lower Bound	Upper Bound
*k*_*1*_ *(i*.*e*., *k*_*on*,*UTP*_*)*	100 μM^-1^ s^-1^	--	--
*k*_*2*_ *(i*.*e*., *k*_*translocation*_*)*	124 s^-1^	51 s^-1^	411 s^-1^
*k*_*3*_ *(i*.*e*., *k*_*UMP incorporation*_*)*	68 s^-1^	--	--
*k*_*4*_ *(i*.*e*., *k*_*off*,*PP*_*)*	10 s^-1^	8 s^-1^	12 s^-1^

The observable output expression is defined as (a*PP_i_ + b) in the fitting routine where only free pyrophosphate is monitored. *k*_*1*_ and *k*_*3*_ values were not optimized in the fitting routine.

### Scheme 1


$$EC9\overset{k_{1}\left[UTP\right]}{\to }EC9.UTP\overset{k_{2}}{\to }EC9\prime .UTP\overset{k_{3}}{\to }EC10.PP_{i}\overset{k_{4}}{\to }EC10+PP_{i}$$

#### Steps for scheme 1: Minimal model for a single round of the NAC with UMP incorporation followed by pyrophosphate release

UTP binds to the EC prior to translocation of the core polymerase. The first step is the binding of the nucleotide to the pre-translocated state of the EC. This is followed in the second step by translocation of the core polymerase to the post-translocated state indicated by EC9’. Step 3 corresponds to nucleotide incorporation in which the 9mer RNA is converted to a 10mer. In step 4, pyrophosphate is released.

### The stopped flow kinetic data set for pyrophosphate release is not consistent with a model in which the slow release of pyrophosphate is due to a slow rate of nucleotide incorporation during one round of the NAC

**Fig 3:**
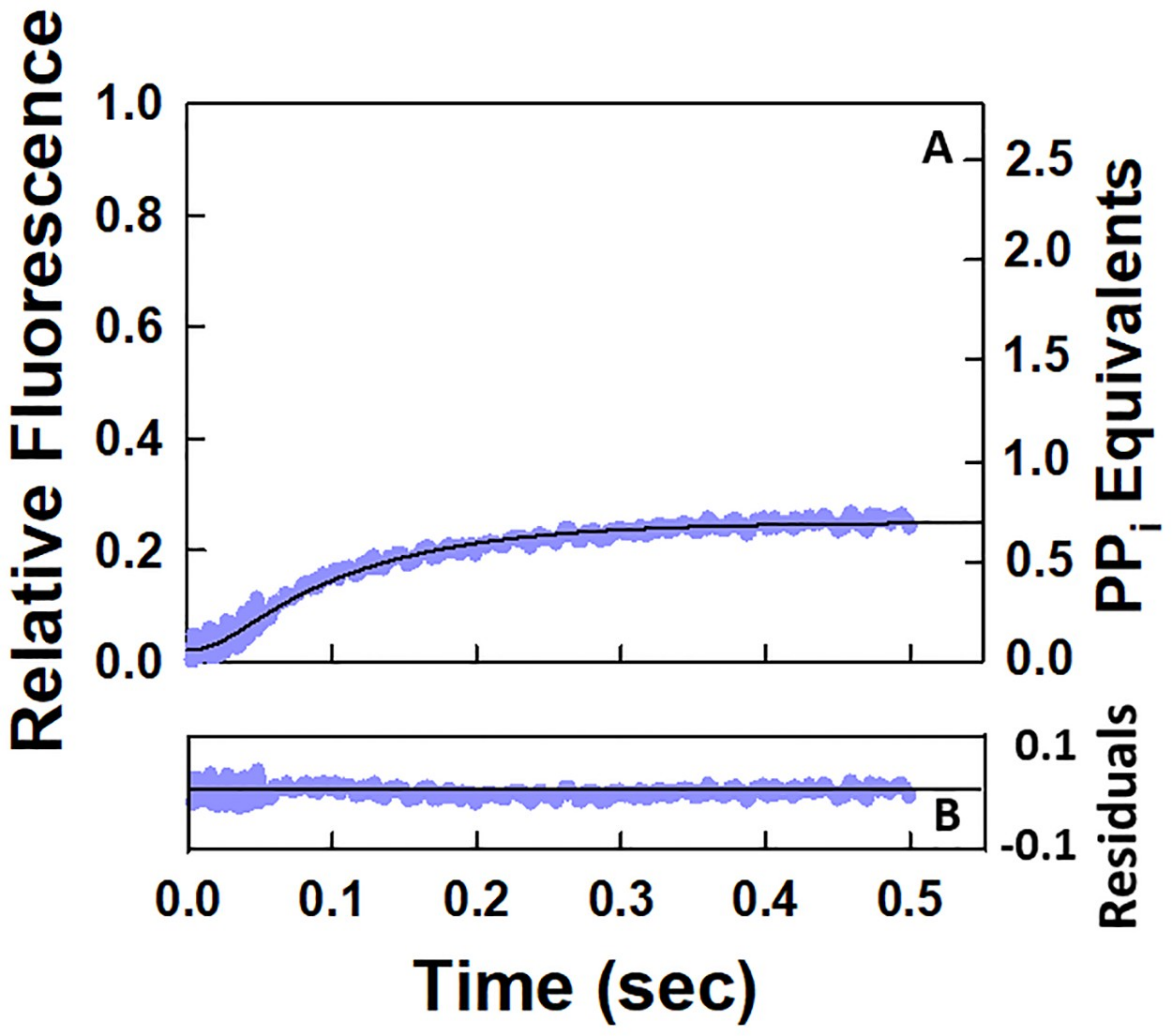
Analysis of the stopped flow kinetic data set for a single round of the NAC assuming that nucleotide incorporation is the rate limiting step in the cycle. (A) The data set shown is the same as that given in Fig 2A for a single round of the NAC at 25°C. The solid black line through the data points corresponds to the fit to Scheme 1 when the value of the rate constant for nucleotide incorporation was set equal to 10 s^-1^. (B) Residual plot for the fit of the data.

To test the hypothesis that a slow rate of nucleotide incorporation limits the rate of pyrophosphate release during a single round of the NAC, the value of the rate constant for nucleotide incorporation was fixed at 10 s^-1^ and the data set in Fig 2A was reanalyzed by using Scheme 1. The results of the fit are given in Fig 3A, and the values of the various rate constants are listed in Table 2. Although the residuals in Fig 3B indicate a good fit, the values of the rate constants for *k*_*translocation*_ and *k*_*off*,*PP*_ are not well-constrained (Table 2). As a result, the stopped flow kinetic data set for the pyrophosphate release curve during one round of the NAC does not support a model in which the rate of incorporation of the nucleotide limits the rate of release of pyrophosphate.

**Table 2:** Values and limits of rate constants for *E*. *coli* RNA polymerase during one round of the NAC based on the model given in Scheme 1 for UTP binding to the pre-translocated state as monitored in stopped flow kinetic studies. Nucleotide incorporation is assumed to be the rate limiting step.

Parameter	Value	Lower Bound	Upper Bound
*k*_*1*_ *(i*.*e*., *k*_*on*,*UTP*_*)*	100 μM^-1^ s^-1^	--	--
*k*_*2*_ *(i*.*e*., *k*_*translocation*_*)*	91 s^-1^	37 s^-1^	8 x 10^8^ s^-1^
*k*_*3*_ *(i*.*e*., *k*_*UMP incorporation*_*)*	10 s^-1^	--	--
*k*_*4*_ *(i*.*e*., *k*_*off*,*PP*_*)*	90 s^-1^	37 s^-1^	8 x 10^8^ s^-1^

The observable output expression is defined as (a*PP_i_ + b) in the fitting routine where only free pyrophosphate is monitored. The values of *k*_*1*_ and *k*_*3*_ values were not optimized in the fitting routine.

### The stopped flow kinetic data set for pyrophosphate release is not consistent with a model for one round of the NAC in which nucleotide binding occurs after translocation of the core polymerase from the pre- to the post-translocated state

**Fig 4:**
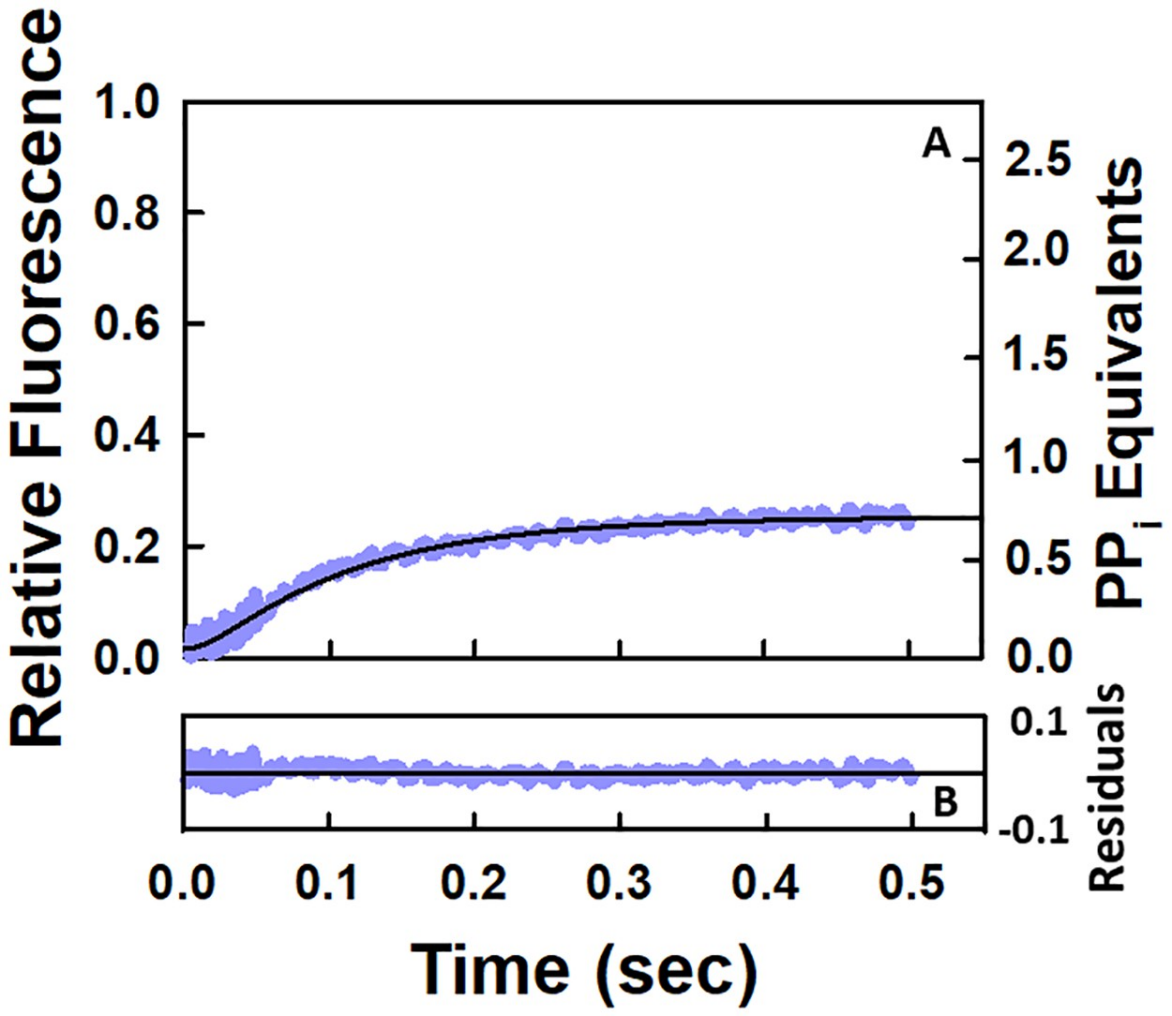
Stopped-flow kinetic results for pyrophosphate release during one round of the NAC with the EC undergoing translocation prior to NTP binding. (A) The data set shown is the same as that given in Fig 2A for a single round of NAC at 25°C. The reaction was monitored by using a coupled enzyme assay as outlined in the experimental section. The solid black line through the data points corresponds to the fit to Scheme 2. (B) Residual plot for the fit of the data.

The model given in Scheme 1 corresponds to a system in which the nucleotide binds to the elongation complex that is in the pre-translocated state. However, the currently accepted classical model for this process involves the initial binding of the nucleotide to the elongation complex in the post-translocated state [11–17]. This model is given in Scheme 2. The data set that was fitted to this model corresponds to that given in Fig 2A and is replotted in Fig 4A along with the fitted line given in black. The values of the corresponding rate constants along with their upper and lower bounds are given in Table 3. Although the residuals in Fig 4B suggest a good fit, the value of the rate constant for *k*_*translocation*_ is not well-constrained. As a result, the stopped flow kinetic data set for the pyrophosphate release curve during one round of the NAC does not support a mechanism in which the nucleotide binds initially to the elongation complex that is in the post-translocated state.

**Table 3:** Values and limits of rate constants for *E*. *coli* RNA polymerase during one round of the NAC based on the model given in Scheme 2 showing NTP binding to the post-translocated state as monitored in stopped flow kinetic studies.

Parameter	Value	Lower Bound	Upper Bound
*k*_*1*_ *(i*.*e*., *k*_*translocation*_*)*	70 s^-1^	0 s^-1^	1x10^8^ s^-1^
*k*_*2*_ *(i*.*e*., *k*_*on*,*UTP*_*)*	100 μM^-1^ s^-1^	--	--
*k*_*3*_ *(i*.*e*., *k*_*UMP incorporation*_*)*	68 s^-1^	--	--
*k*_*4*_ *(i*.*e*., *k*_*off*,*PP*_*)*	10 s^-1^	8 s^-1^	11 s^-1^

The observable output expression is defined as (a*PP_i_ + b) in the fitting routine where only free pyrophosphate is monitored. *K*_*2*_ and *k*_*3*_ values were not optimized in the fitting routine. The value of *k*_*UMP incorporation*_ was set equal to the corresponding value listed in Table 1.

### Scheme 2


$$EC9\overset{k_{1}}{\to }EC9\prime \overset{k_{2}\left[UTP\right]}{\to }EC9\prime .UTP\overset{k_{3}}{\to }EC10.PP_{i}\;\overset{k_{4}}{\to }EC10\;+\;PP_{i}$$

#### Steps for scheme 2: Minimal model for a single round of the NAC with UMP incorporation followed by pyrophosphate release

UTP binds to the EC in the post-translocated state. The first step is the translocation of the enzyme from the pre- to the post-translocated state. The post-translocated state is represented by EC9’. This is followed by the binding of UTP to EC9’. The third step corresponds to nucleotide incorporation with the RNA going from a 9mer to a 10mer. The final step is pyrophosphate release.

### Embedded in the data reporting to show rapid pyrophosphate release from RNA polymerase (*k*_*off*,*PP*_ = 104 s^-1^ after CMP incorporation) is a region that is consistent with slow pyrophosphate release (*k*_*off*,*PP*_ = 4 s^-1^) during a single round of the NAC

**Fig 5:**
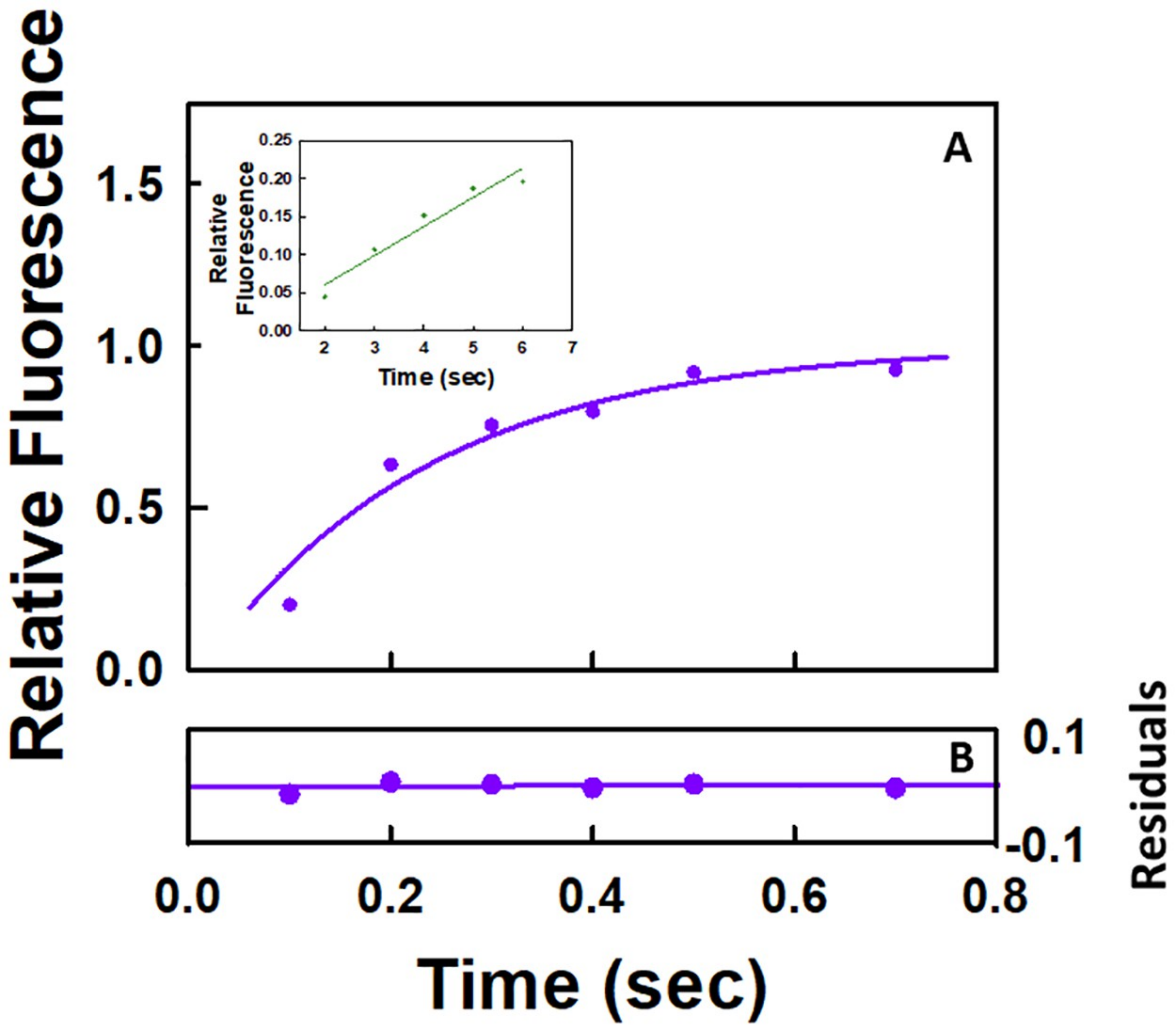
Analysis of the pyrophosphate release curve over the region of 0.08 to 0.7 sec from the study reporting rapid pyrophosphate release from the EC during a single round of the NAC following CMP incorporation. **(A)** The concentration of the EC after mixing was 0.2 μM and the CTP final concentration after mixing was 200 μM. The line through the data points corresponds to the fit to Scheme 3. **(B)** Residual plot for the fit of the data. **(Inset)** Variation of the pyrophosphate release curve over the range of 1 to 7 seconds along with a linear fit to the data.

In a previous study [41], results were presented supporting the hypothesis that pyrophosphate release after nucleotide incorporation is rapid (*i*.*e*., 82–133 s^-1^) during a single round of the NAC [41]. Inspection of the semi-logarithmic plot of normalized fluorescence versus time for pyrophosphate release in the previous study (Fig S3A [41]) shows that it is multiphasic. A lag phase is followed by a rapid phase that goes approximately from 0.01 to 0.08 seconds, an intermediate phase that goes approximately from 0.08 to 0.7 seconds and the final slow phase that goes approximately from 1.0 to 10 seconds. A screenshot was used to capture the semi-logarithmic plot and the intermediate phase between 0.08 and 0.7 seconds was analyzed. A calculation based on the reported rate constant of 104 s^-1^ for rapid pyrophosphate release indicates that the reaction occurring in the rapid phase is over in approximately 0.05 seconds. As a result, it does not interfere with the analysis of the data between 0.08 and 0.7 seconds. Data points over the range of 0.08 and 0.7 as well as from 1 and 7 seconds were extracted as outlined in the methods section. There is a gradual linear increase in fluorescence with time for the data points over the range of 1 to 7 seconds (Fig 5 Inset). The value of the rate constant corresponding to this region of the pyrophosphate release curve is 0.04 s^-1^. Using this rate constant, the values of the fluorescence over the range of 0.08 and 0.7 seconds due to the background reaction can be determined. This was then subtracted from the data collected from 0.8 to 0.7 seconds. The plot of the corrected values over the range of 0.08 and 0.7 seconds is given Fig 5A along with a theoretical fit based on Scheme 3. A three-step rather than a four-step scheme was used because there was an insufficient number of data points to resolve the values of the rate constants for a four-step model. The values of the various parameters are given in Table 4. The goodness of the fit based on the residuals (Fig 5B) and the well-constrained value of *k*_*off*,*PP*_ (Table 4) is consistent with the three-step model in Scheme 3. The value of *k*_*off*,*PP*_ (4 s^-1^, Table 4) is comparable to the value obtained in the current study for the slow release of pyrophosphate from RNA polymerase (3–10 s^-1^, Tables 1, 5–7 and 9). The value of *k*_*off*,*PP*_ (4 s^-1^) is approximately 20 times less than the reported value for CMP incorporation (*k*_*CMP incorporated*_ = 82 s^-1^). It should be noted that the value of *k*_*off*,*PP*_ (4 s^-1^) is the same as that determined without correction for the background reaction. This is to be expected because the value of *k*_*off*,*PP*_ is 100 times greater than the rate constant for the background reaction.

**Table 4:** Values and limits of rate constants for *E*. *coli* RNA core polymerase during one round of the NAC based on the model given in Scheme 3 for the incorporation of CMP and the release of pyrophosphate.

Parameter	Value	Lower Bound	Upper Bound
*k*_*1*_ *(i*.*e*., *k*_*on*,*CTP*_*)*	100 μM^-1^ s^-1^	--	--
*k*_*2*_ *(i*.*e*., *k*_*CMP incorporation*_*)*	81 s^-1^	--	--
*k*_*3*_ *(i*.*e*., *k*_*off*,*PP*_*)*	4 s^-1^	3 s^-1^	4 s^-1^

The observable output expression is defined as (a*PP_i_ + b) in the fitting routine where only free pyrophosphate is monitored. *k*_*1*_ and *k*_*2*_ values were not optimized in the fitting routine. The value of *k*_*CMP incorporation*_ is the one reported in their study [41].

**Table 5:** Values and limits of rate constants for *E*. *coli* RNA polymerase during two rounds of the NAC based on the model given in Scheme 4 as monitored in stopped flow kinetic studies.

Parameter	Value	Lower Bound	Upper Bound
*k*_*1*_ *(i*.*e*., *k*_*on*,*UTP*_*)*	100 μM^-1^ s^-1^	--	--
*k*_*2*_ *(i*.*e*., *k*_*translocation*_*)*	197 s^-1^	187	412
*k*_*3*_ *(i*.*e*., *k*_*UMP incorporation*_*)*	68 s^-1^	--	--
*k*_*4*_ *(i*.*e*., *k*_*on*,*ATP*_*)*	100 μM^-1^ s^-1^	--	--
*k*_*5*_ *(i*.*e*., *k*_*composite*_*)*	18 s^-1^	13	21
*k*_*6*_ *(i*.*e*., *k*_*off*,*PP*_*)*	3 s^-1^	1	3

The observable output expression is defined as (a*PP_i_ + b) in the fitting routine where only free pyrophosphate is monitored. *k*_*1*_, *k*_*3*_ and *k*_*4*_ values were not optimized in the fitting routine. The value of *k*_*UMP incorporation*_ was set equal to the corresponding value listed in Table 1.

**Table 6:** Values and limits of rate constants for *E*. *coli* RNA polymerase in the presence of the cognate first NTP (UTP) and noncognate second NTPs (CTP and GTP), respectively, for incorporation during one round of the NAC based on the model given in Scheme 1 for NTP binding to the pre-translocated state as monitored in stopped flow kinetic studies.

	Parameter	Value	Lower Bound	Upper Bound
	*k*_*1*_ *(i*.*e*., *k*_*on*,*UTP*_*)*	100 μM^-1^ s^-1^	--	--
Noncognate NTP				
CTP	*k*_*2*_ *(i*.*e*., *k*_*translocation*_*)*	157 s^-1^	80 s^-1^	388 s^-1^
GTP	*k*_*2*_ *(i*.*e*., *k*_*translocation*_*)*	70 s^-1^	16 s^-1^	249 s^-1^
Noncognate NTP				
CTP	*k*_*3*_ *(i*.*e*.,*k*_*UMP incorporation*_*)*	72 s^-1^	--	--
GTP	*k*_*3*_ *(i*.*e*.,*k*_*UMP incorporation*_*)*	73 s^-1^	--	--
Noncognate NTP				
CTP	*k*_*4*_ *(i*.*e*., *k*_*off*,*PP*_*)*	8 s^-1^	7 s^-1^	8 s^-1^
GTP	*k*_*4*_ *(i*.*e*., *k*_*off*,*PP*_*)*	10 s^-1^	7 s^-1^	23 s^-1^

The observable output expression is defined as (a*PP_i_ + b) in the fitting routine where only free pyrophosphate is monitored. *k*_*1*_ and *k*_*3*_ values were not optimized in the fitting routine.

**Table 7:** Values of rate constants for *E*. *coli* core RNA polymerase during one round of the NAC in the presence of [γ-^32^p]ATP based on the model given in Scheme 5 for ATP binding to the pre-translocated state as monitored in QF-TLC studies.

Parameter	Value	Lower Bound	Upper Bound
*k*_*1*_ *(i*.*e*., *k*_*on*,*ATP*_*)*	100 μM^-1^ s^-1^	--	--
*k*_*2*_ *(i*.*e*., *k*_*translocation*_*)*	137 s^-1^	--	--
*k*_*3*_ *(i*.*e*., *k*_*AMP incorporation*_*)*	70 s^-1^	--	--
*k*_*4*_ *(i*.*e*., *k*_*off*,*PP*_*)*	3 s^-1^	1 s^-1^	3 s^-1^

The observable output expression is defined as (a*PP_i_ + b) in the fitting routine where only free pyrophosphate is monitored. The estimate for *k*_*translocation*_ is the average of the four values for this rate constant given in Tables 1, 5 and 6 and the estimate for *k*_*AMP incorporation*_ is approximated by the average of the four values of *k*_*UMP incorporation*_ given in Tables 1, 5 and 6. Only *k*_*4*_ was optimized in the fitting routine.

### Scheme 3


$$EC16\overset{k_{1}\left[CTP\right]}{\to }EC16.CTP\overset{k_{2}}{\to }EC17.PP_{i}\overset{k_{3}}{\to }EC17+\;PP_{i}$$

#### Steps for scheme 3: Minimal model for a single round of the NAC with CMP incorporation followed by pyrophosphate release

The first step is the binding of CTP to EC16. This is followed by nucleotide incorporation with the RNA going from a 16mer to a 17mer in the second step. The final step is pyrophosphate release.

Although both studies used the same coupled enzyme assay involving PPase (pyrophosphatase) and MDCC-PBP, there were major differences between the two protocols. As outlined in the methods section of the current study, both phosphate and pyrophosphate mops were used to eliminate the respective contaminants from all the reaction mixtures. A phosphate mop was also used to purge the lines of the stopped flow apparatus. This was done to ensure that there were no other sources of phosphate or pyrophosphate in the system that could be mistaken for pyrophosphate generated by the reaction catalyzed by RNA polymerase. In the other study [41], a pyrophosphate mop was not used on any of the solutions and a phosphate mop was only used to eliminate phosphate from the stock nucleotide solutions and to purge the lines of the stopped flow apparatus. This means that the nucleotide stock solutions contained pyrophosphate as a contaminant. If the stock nucleotide solutions that had been treated with the phosphate mop were subjected to multiple thawing and freezing cycles without additional treatments with the phosphate mop, then there would have been a significant increase in the concentration of contaminating phosphate over time [42]. By not using a pyrophosphate mop and not explicitly indicating whether the stock nucleotide solutions had been retreated with a phosphate mop, the authors of the other study cannot unequivocally state that the increase in the fluorescence change over the time range of 0.01 and 0.08 seconds was due exclusively to the reaction catalyzed by RNA polymerase.

**Fig 6:**
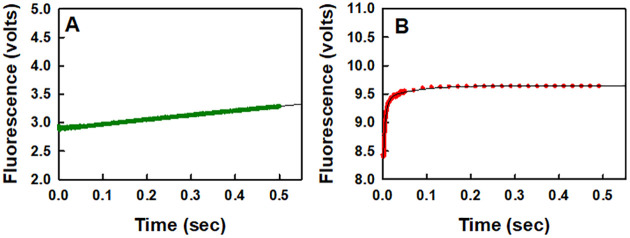
Efficacies of using phosphate and pyrophosphate mops together (A) or a phosphate mop alone (B) to eliminate background fluorescence due to the corresponding contaminants present in the nucleotide samples. The final concentration of UTP after mixing in the stopped-flow apparatus in each case was 50 μM. The protocol for conducting this study is the same as that given in the methods section except that the elongation complex is not present.

The efficacy of using a pyrophosphate mop along with one for phosphate is illustrated in Fig 6. In the control experiment set up with both phosphate and pyrophosphate mops present along with nucleotides but no elongation complex, there was a gradual linear increase in the fluorescence with time (Fig 6A). The value of the rate constant for this change is 0.8 s^-1^. The origin of this change is not clear. It may be a side reaction of PPase that occurs with nucleotides. In the control experiment set up to indicate what happens when just a phosphate mop was present along with nucleotides in the absence of the elongation complex, there was a large perturbation in the fluorescence that is due apparently to contaminating pyrophosphate in the nucleotide sample (Fig 6B). A single exponential fit of the data led to a value of the observed first order rate constant of 160±3 s^-1^.

**Fig 7:**
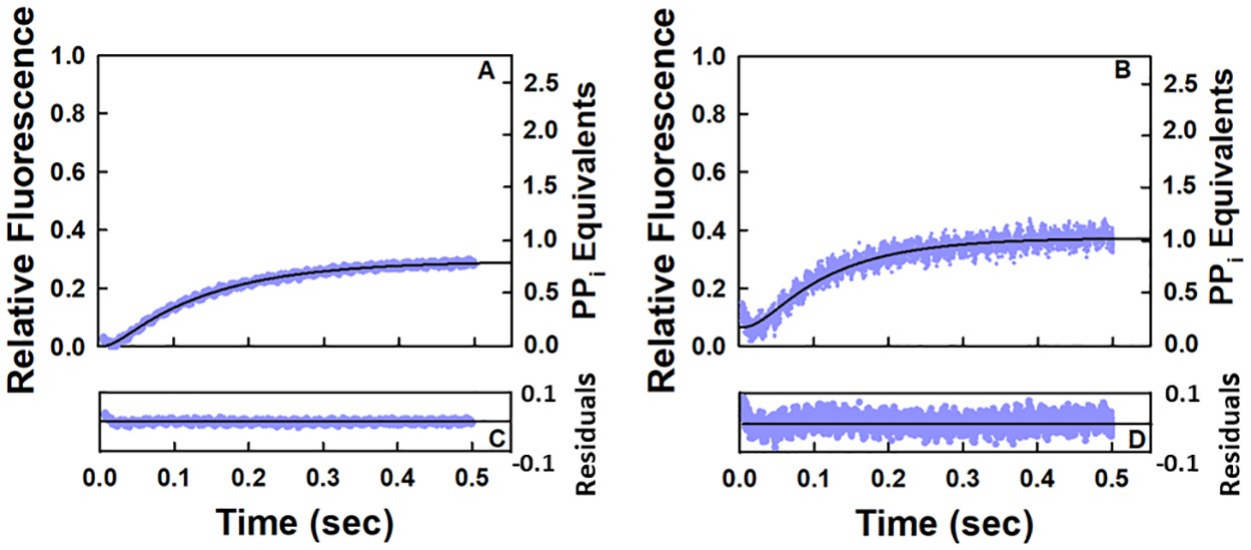
Stopped-flow kinetic results for pyrophosphate release in the presence of UTP and CTP (A) as well as UTP and GTP (B). NTP concentrations were each 50 μM with EC concentration of 0.2 μM after mixing at 25°C. The appropriate control in the absence of the EC corresponding to the background was subtracted in each case. The pyrophosphate release curve for UTP and CTP corresponds to the average of four runs and the curve for UTP and GTP corresponds to the average of two runs. The solid black lines through the data points correspond to the fits to Scheme 1.

A functional assay as outlined in detail in the methods section was used in the current study to establish that the pyrophosphate release curves represent pyrophosphate generated by the reaction catalyzed by RNA polymerase. The amplitude at the endpoint of the curve for incorporation of two nucleotides should be twice that observed for incorporation of a single nucleotide. Four equations as indicated in the methods section can be generated for the respective amplitudes for incorporation of two nucleotides (UMP and AMP), one nucleotide (UMP) and one nucleotide in the presence of the corresponding noncognate nucleotides (UMP/CTP as well as UMP/GTP). From these four equations, the value of the relative fluorescence corresponding to one pyrophosphate equivalent can be calculated and used to plot the variation of pyrophosphate equivalents generated as a function of time. As can be seen in Fig 1, the ratio of pyrophosphate equivalents generated during one and two rounds of the NAC is approximately two. Moreover, as is given in Fig 7, the pyrophosphate equivalent generated in the presence of one cognate and one noncognate nucleotide extrapolates in both cases to approximately one. This latter result indicates that the pyrophosphate release curve is not due to pyrophosphate contaminants in the nucleotide solutions that increase by a factor of approximately two when the total nucleotide concentration is doubled. Functional assays have been used previously to demonstrate that the pyrophosphate release curve as measured in a coupled enzyme reaction involving pyrophosphatase and a fluorescently labeled phosphate binding protein is due to the reaction catalyzed by the enzyme in question [43].

### The rate of release of the first pyrophosphate equivalent is increased in the presence of the next nucleotide for incorporation during two rounds of the NAC

UMP incorporation is followed by incorporation of AMP during two rounds of the NAC for the elongation complex composed of the core polymerase and the nucleic acid scaffold given in Fig 1A containing the 9mer RNA. Pyrophosphate release occurs prior to nucleotide incorporation only during the second round of the NAC. The pyrophosphate release curve for two rounds of the NAC is given in Fig 2B. The data set was fitted to the model in Scheme 4 and the values of the respective rate constants are given in Table 5. The goodness of the fit based on the residuals (Fig 2D) and the well-constrained values of the various rate constants (Table 5) supports the six-step model in Scheme 4. In the formulation of Scheme 4, three processes were combined in step 5 leading to a composite rate constant. These three processes include release of the first pyrophosphate equivalent, translocation of the core polymerase and incorporation of the second nucleotide (AMP). One of the processes in the triad in essence limits the rates of the other two processes. Based on the values of the rate constants listed in Table 1, the rate limiting process in step 5 is most likely the release of the first pyrophosphate equivalent. All the other processes in the triad can only proceed as fast as the slowest one. The effects of the first pyrophosphate equivalent as well as the second nucleotide for incorporation on the second round of the NAC can be investigated based on the results obtained for one and two rounds of the NAC. The binding of the next nucleotide for incorporation resulted in an increase in the rate of release of the first pyrophosphate equivalent by a factor of approximately two (10 s^-s^, Table 1 versus 18 s^-1^, Table 5). The presence of the first pyrophosphate equivalent bound to the elongation complex suppressed the rate of translocation by a factor of approximately 7 (124 s^-1^, Table 1 versus 18 s^-1^, Table 5). The presence of the first pyrophosphate equivalent bound at the active site also suppressed the rate of nucleotide incorporation by a factor of approximately four (68 s^-1^; Table 1 versus 18 s^-1^, Table 5).

### Scheme 4


$$EC9\overset{k_{1}\left[UTP\right]}{\to }\;E9.UTP\overset{k_{2}}{\to }\;E9\prime .UTP\overset{k_{3}}{\to }\;EC10.PP_{i}\overset{k_{4}\left[ATP\right]}{\to }EC10.PP_{i}.ATP\overset{k_{5}}{\to }EC11.PP_{i}+PP_{i}\overset{k_{6}}{\to }EC11+PP_{i}$$

#### Steps for scheme 4: Minimal model for two rounds of the NAC

The successive incorporations of UMP followed by AMP are accompanied by pyrophosphate release after each incorporation. The first step is the binding of UTP to the pre-translocated state of EC9. This is followed in the second step by the translocation of the core polymerase to the post-translocated state. The post-translocated state is represented by EC9’. This translocation is accompanied by the entry of the nucleotide into the active site. The third step corresponds to nucleotide incorporation in which the 9mer RNA is converted to an 10mer. ***PP***_***i***_ represents the first pyrophosphate equivalent. In the fourth step, the second nucleotide (ATP) for incorporation binds to EC10.**PP**_**i**_ in the pre-translocated state. The fifth step is treated as a composite of three processes involving release of the first pyrophosphate equivalent, translocation of the core polymerase and nucleotide (AMP) incorporation. The sixth step corresponds to the release of the second pyrophosphate equivalent.

### The enhancement in the rate of release of the first pyrophosphate equivalent during two rounds of the NAC requires the cognate nucleotide for incorporation

The pyrophosphate release curves in the presence of UTP with either CTP or GTP, respectively, are given in Fig 7. Because there was no indication of rapid pyrophosphate release in the presence of the noncognate nucleotides, each data set was fitted to Scheme 1. The reported value of the rate constant *(k*_*2*_*)* for UMP incorporation from quench-flow studies is 58 s^-1^ for this EC [40]. Using this as a starting point, the value of *k*_*2*_ was varied to optimize the fit for the stopped-flow data according to the model in Scheme 1. The goodness of each fit based on the residuals (Fig 7C and 7D) and the well-constrained values of the various rate constants (Table 6) support the four-step model in Scheme 1 for one round of the NAC as monitored by the pyrophosphate release curve. The values of the rate constant for *k*_*UMP incorporation*_ (72 and 73 s^-1^; Table 6) in the presence of the noncognate nucleotides are comparable to the value of the rate constant in their absence (68 s^-1^; Table 1). The values for *k*_*off*,*PP*_ given in Table 6 (8 and 10 s^-1^) are comparable to that observed in the presence of only UTP (10 s^-1^; Table 1). There is also reasonable agreement with respect to *k*_*translocation*_ (124 s^-1^; Table 1) with values of 70 and 157 s^-1^ (Table 6).

### Quench flow-TLC (QF-TLC) studies support the stopped-flow kinetic results indicating that pyrophosphate release after one round of the NAC is slow relative to nucleotide incorporation and that the second nucleotide for incorporation increases the rate of release of the first pyrophosphate equivalent

Studies on poliovirus RNA-dependent RNA polymerase (3D^pol^) [44] served as the precedent for using quench flow and thin layer chromatography in the investigation of the NAC of *E*. *coli* RNA polymerase. In a combination of experiments involving [^32^P]PP_i_ and [γ-^32^P]ATP, pyrophosphate exchange was investigated in the kinetic analysis of ribonucleotide incorporation by 3D^pol^ [44]. In the current study, the QF-TLC technique is used to monitor [^32^P]PP_i_ release after one and two rounds of the NAC as catalyzed by RNA polymerase. For a single round of the NAC, only [γ-^32^P]ATP was present. For two rounds of the NAC, [γ-^32^P]ATP was present along with the next nucleotide (*i*.*e*., CTP) for incorporation.

The QF-TLC studies complement the stopped flow kinetic studies for monitoring pyrophosphate release during the NAC. The only enzyme present in the QF-TLC studies is RNA polymerase, whereas the stopped flow kinetic studies required two other enzymes to couple pyrophosphate release from RNA polymerase to a system that gives rise to a change in fluorescence. If the results obtained from these two techniques are comparable, this eliminates the possibility that RNA polymerase is not the rate limiting enzyme in the coupled enzyme assay used in the stopped flow experiments. The QF-TLC method also provided a means of directly comparing pyrophosphate release during one round of the NAC to the release of the first pyrophosphate equivalent during two rounds of the NAC. In contrast, the stopped flow kinetic analysis during two rounds of the NAC required modelling to resolve the values of the rate constants for the release of the two pyrophosphate equivalents. One drawback to the QF-TLC approach is that it does not allow for robust fitting of data to a model because there are far fewer data points than in the case of the stopped flow kinetic studies. The QF-TLC studies were conducted by using a 10mer RNA primer instead of the 9mer primer used in the stopped-flow kinetic studies because only [γ-^32^P] purine nucleotides are commercially available. The nucleic acid scaffold for the QF-TLC studies is given in Fig 1B. The sequences of the template and non-template strands of the nucleic acid scaffold were altered as shown in Fig 1B to limit the reaction to just two rounds of the NAC in the presence of [γ-^32^P]ATP and CTP.

In the QF-TLC studies, the reaction catalyzed by RNA polymerase can be quenched (stopped) by using either EDTA or HCl. In previous studies, it was observed that EDTA quenching of the reaction catalyzed by RNA polymerase leads to values of the rate constant for nucleotide addition that are significantly greater than those obtained when using HCl to quench [45, 46]. This can be explained by the observation that the active site closes upon nucleotide binding [5]. In this state, nothing gets in or out of the active site. HCl quenches the reaction by denaturing RNA polymerase whether the active site is closed or open. In contrast, EDTA is thought to inhibit the reaction by removing Mg^2+^ ions from unbound nucleotide substrate molecules. This suppresses the NAC because RNA polymerase requires two Mg^2+^ ions at the active site to display activity. One of the Mg^2+^ ions is tightly bound at the active site. The second one enters the active site bound to the nucleotide substrate. In the presence of high levels of EDTA, the Mg^2+^ is removed from the free nucleotide molecules and therefore they cannot deliver the second required Mg^2+^. Any nucleotide molecules sequestered in the active site prior to the addition of EDTA go on to be incorporated into the RNA. EDTA was used in the QF-TLC studies because pyrophosphate release and not its formation is being monitored.

**Fig 8:**
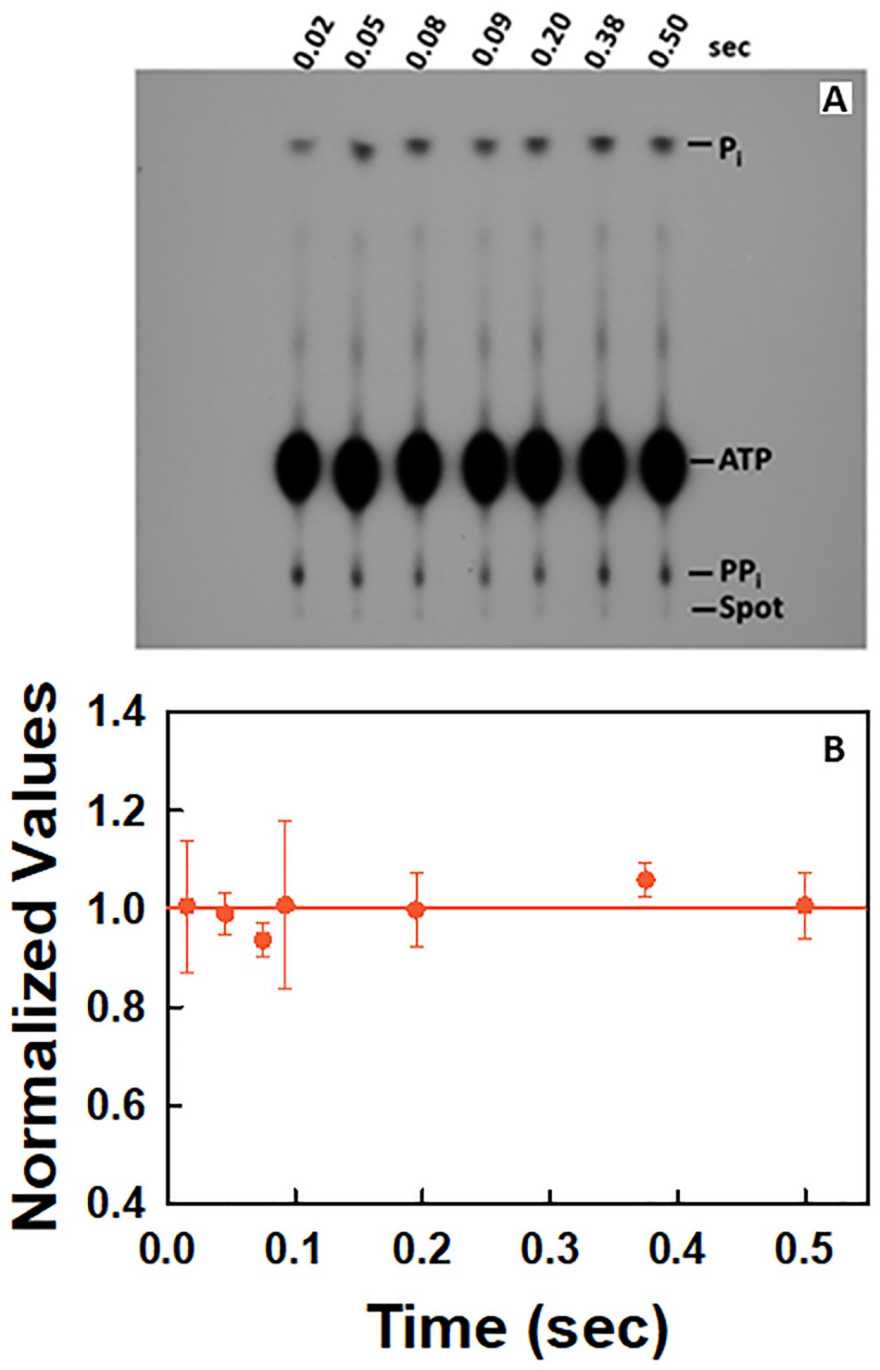
Control for QF-TLC studies illustrating the purity of [γ-^32^P]ATP and that there is no time-dependent variation in the intensity of the band corresponding to [γ-^32^P]PP_i_. **(A)** In the autoradiogram, each lane corresponds to a quench at the indicated time. The concentration of [γ-^32^P]ATP [60 μCi/pmol] after mixing was 50 μM. A duplicate is given in S1 Raw images along with a larger version of the image in Fig 8A. (**B)** Plot of the intensity of the [^32^P]PP_i_ contaminant spot over time. The data correspond to the average of two independent experiments. For analysis, each data set was normalized by dividing the intensity of each spot by the average of the intensities for the seven time points. The normalized values of the two sets of data were then averaged and the standard deviation of each time point was determined.

To ascertain the purity of the [γ-^32^P]ATP sample, controls were performed as a function of time in the absence of the elongation complex (Fig 8). Assignments of the spots agree with published studies [44, 47]. The [γ-^32^P]ATP samples contained both [^32^P]P_i_ and [^32^P]PP_i_ contaminants (Fig 8A). Studies were also conducted to determine if there is any variation in the amount of [^32^P]PP_i_ contaminant present as a function of time. The intensity of the band corresponding to [^32^P]PP_i_ did not vary as a function of time (Fig 8B). Unlike in the case of the stopped flow kinetic studies in which pyrophosphate contaminants could be converted to phosphate and give rise to an increase in the fluorescence upon binding to PBP-MDCC, the [^32^P]PP_i_ contaminant simply shifted the baseline of the experiments conducted in the presence of the elongation complexes.

**Fig 9:**
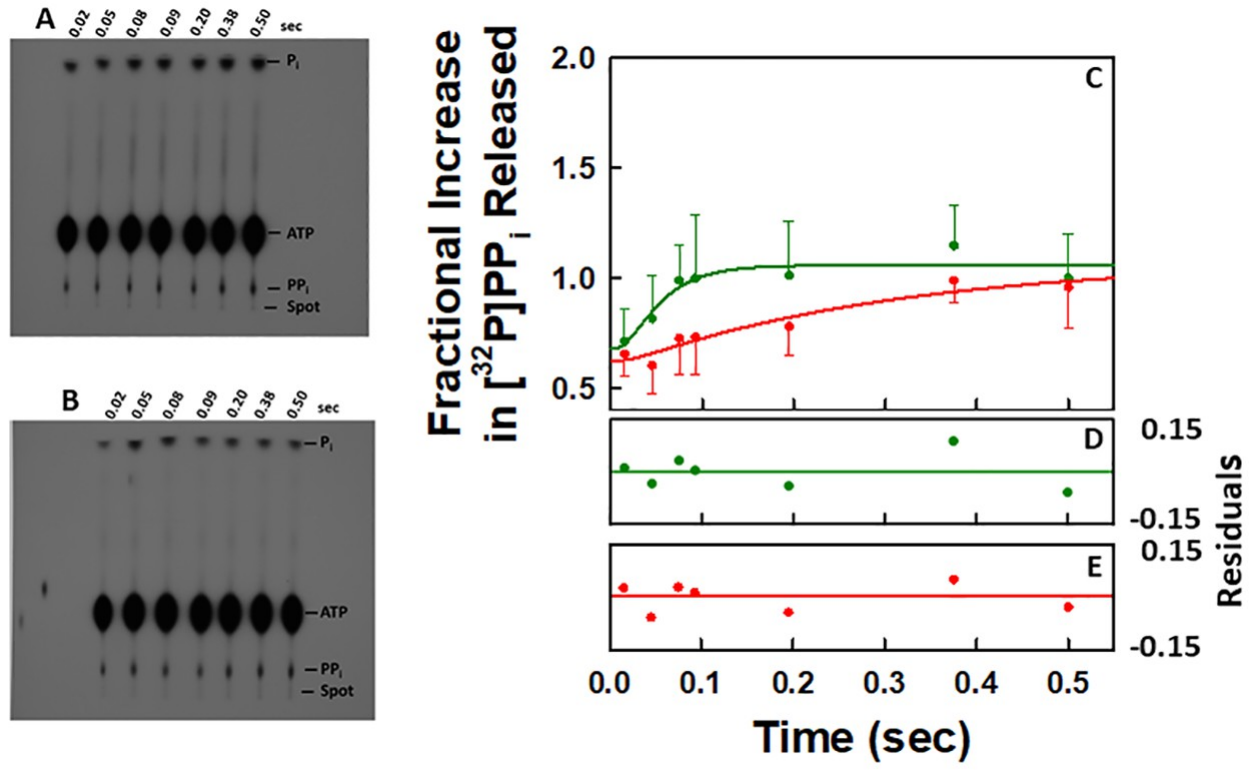
QF-TLC analyses of pyrophosphate release during one and two rounds of the NAC. The autoradiograms correspond to the time-dependent release of [^32^P]PP_i_ in the presence of (**A**) 0.5 μM EC and 50 μM [γ-^32^P]ATP [60 μCi/pmol] or (**B**) 0.5 μM EC with 50 μM [γ-^32^P]ATP [60 μCi/pmol] and 50 μM CTP, respectively, after mixing at 25°C. (**C**) Averages for each data set in C were obtained as indicated in Methods. The non-zero y-intercept in each case is due to contaminating [^32^P]PP_i_ in the reaction mixtures. (●) Plot of the time dependent release of [^32^P]PP_i_ in the presence of 0.5 μM EC and 50 μM [γ-^32^P]ATP [60 μCi/pmol] after mixing. The data correspond to the average of four independent experiments. The line through the data points was generated by fitting the data to the model in Scheme 5. (●) Plot of the time dependent release of [^32^P]PP_i_ in the presence of 0.5 μM EC, 50 μM [γ-^32^P]ATP [60 μCi/pmol] and 50 μM CTP after mixing at 25°C. The data correspond to the average of four independent experiments. The line through the data points was generated by fitting the data to the model in Scheme 6. **(E)** Residual plot for the fit of the data for one round of the NAC. **(D)** Residual plot for the fit of the data for two rounds of the NAC.

An example of the autoradiogram obtained for the time dependent release of [^32^P]PP_i_ from the EC upon AMP incorporation in the presence of [γ-^32^P]ATP during one round of the NAC is given in Fig 9A. A plot of the time dependent release of [^32^P]PP_i_ during one round of the NAC is given in Fig 9C (●). It is the average of four independent experiments. The autoradiograms of the other three experiments are given in S1 Raw images along with a larger version of the image in Fig 9A. It is apparent from the data points that the reaction is close to completion in about 0.5 sec.

An example of the autoradiogram obtained for the time dependent release of [^32^P]PP_i_ from the EC after AMP incorporation in the presence of [γ-^32^P]ATP and CTP is given in Fig 9B. A plot of the time dependent release of [^32^P]PP_i_ from the EC during two rounds of the NAC is given in Fig 9C (●). It is the average of four independent experiments. The other three autoradiograms are given in S1 Raw images along with a larger version of the image in Fig 9B. In the presence of CTP, the release of the first [^32^P]PP_i_ equivalent is over in approximately 0.2 seconds. A comparison of the two data sets in Fig 9C illustrates that the presence of the next cognate nucleotide for incorporation increases the rate of release of the first pyrophosphate equivalent. This agrees with the stopped flow kinetic data.

To determine how closely the results from the QF-TLC data correspond to those obtained from modeling the stopped flow kinetic data, the QF-TLC data sets were analyzed by using KinTek Explorer. Because of the limited number of data points obtained in each QF-TLC experiment, it was not possible to optimize the value for more than one parameter. For the QF-TLC data set for one round of the NAC, Scheme 5 was used to ascertain how well the data corresponded to the model. Only the value of *k*_*off*,*PP*_ was optimized in the fit. The results from the analysis of the QF-TLC data set are illustrated in Fig 9C (●) by the solid red line through the data points and the value of *k*_*off*,*PP*_ was found to equal 3 sec^-1^ (Table 7). There is good agreement between the QF-TLC data and the proposed model [Fig 9C (●)] as indicated by the residuals (Fig 9E) and the constraint of the value of *k*_*off*,*PP*_ (Table 7). The value of the rate constant for pyrophosphate release is consistent with those obtained in the stopped flow kinetic studies for the release of pyrophosphate during one round of the NAC (Tables 1 and 6).

For the QF-TLC data set for two rounds of the NAC, Scheme 6 was used to ascertain how well the data points correspond to the model. The results from the analysis of the QF-TLC data set are illustrated in Fig 9C (●) by the solid green line through the data points and the value of *k*_*composite*_ was found to equal 28 sec^-1^ (Table 8) where *k*_*composite*_ corresponds to the triad of processes involving the release of the first pyrophosphate equivalent, translocation of the core polymerase and incorporation of the second nucleotide. There is good agreement between the data and the proposed model in this case [Fig 9C (●)] as indicated by the residuals (Fig 9D) and the constraint of the value of *k*_*composite*_ (Table 8). The value of the rate constant for *k*_*composite*_ is somewhat greater than the corresponding value obtained in the stopped flow kinetic studies for the release of pyrophosphate in the presence of the next cognate nucleotide for incorporation (18 sec^-1^, Table 5). This may be a sequence dependent phenomenon or just random variation in the value of *k*_*composite*_.

**Table 8:** Values of rate constants for *E*. *coli* core RNA polymerase during two rounds of the NAC in the presence of [γ-^32^P]ATP and CTP as monitored by QF-TLC.

Parameter	Value	Lower Bound	Upper Bound
*k*_*1*_ *(i*.*e*., *k*_*on*,*ATP*_*)*	100 μM^-1^ s^-1^	--	--
*k*_*2*_ *(i*.*e*., *k*_*translocation*_*)*	137 s^-1^	--	--
*k*_*3*_ *(i*.*e*., *k*_*AMP incorporation*_*)*	70 s^-1^	--	--
*k*_*4*_ *(i*.*e*., *k*_*on*,*CTP*_*)*	100 μM^-1^ s^-1^	--	--
*k*_*5*,_ *(i*.*e*., *k*_*composite*_*)*	28 s^-1^	19 s^-1^	28 s^-1^

The observable output expression is defined as (a*PP_i_ + b) in the fitting routine where only free pyrophosphate is monitored. The estimate for *k*_*translocation*_ is the average of the four values for this rate constant given in Tables 1, 5 and 6 and the estimate for *k*_*AMP incorporation*_ is approximated by the average of the four values of *k*_*UMP incorporation*_ given in Tables 1, 5 and 6. Only *k*_*5*_ was optimized in the fitting routine.

### Scheme 5


$$EC10\overset{k_{1}\left[ATP^{32}\right]}{\to }\;EC10.ATP^{32}\;\overset{k_{2}}{\to }EC10\prime .ATP^{32}\;\overset{k_{3}}{\to }EC11.PP_{i}^{32}\;\overset{k_{4}}{\to }EC11\;+\;PP_{i}^{32}$$

#### Steps for scheme 5: Minimal model for a single round of the NAC with AMP incorporation followed by pyrophosphate release as monitored by QF-TLC

ATP binds to the EC prior to translocation of the core polymerase. The first step is the binding of the nucleotide to the EC in the pre-translocated state. This is followed in the second step by translocation of the core polymerase to the post-translocated state indicated by EC10’. Step 3 corresponds to nucleotide incorporation in which the 10mer RNA is converted to an 11mer. In step 4, ^32^PP is released.

### Scheme 6


$$EC10\overset{k_{1}\left[ATP^{32}\right]}{\to }\;EC10.ATP^{32}\overset{k_{2}}{\to }\;EC10\prime .ATP^{32}\overset{k_{3}}{\to }\;EC11.PP_{i}^{32}\overset{k_{4}\left[CTP\right]}{\to }EC11.PP_{i}^{32}.CTP\overset{k_{5}}{\to }EC12.PP_{i}+PP_{i}^{32}$$

#### Steps for scheme 6: Minimal model for two rounds of the NAC with consecutive incorporation of AMP and CMP with release of the first pyrophosphate equivalent as monitored by QF-TLC

The first step is the binding of [γ-^32^P]ATP to the EC in the pre-translocated state. This is followed in the second step by translocation of the core polymerase to the post-translocated state indicated by EC10’. Step 3 corresponds to nucleotide incorporation in which the 10mer RNA is converted to an 11mer with the generation of the first pyrophosphate equivalent bound at the active site. In step 4, CTP binds to the [EC11.^32^PP_i_] complex. Step 5 is formulated as a composite of three processes involving the release of the first pyrophosphate equivalent, translocation of the core polymerase and nucleotide incorporation to convert EC11 to EC12. $PP_{i}^{32}$ corresponds to the first pyrophosphate equivalent.

### QF-TLC studies support the stopped-flow kinetic results indicating that only the cognate nucleotide for incorporation increases the rate of release of the first pyrophosphate equivalent

**Fig 10:**
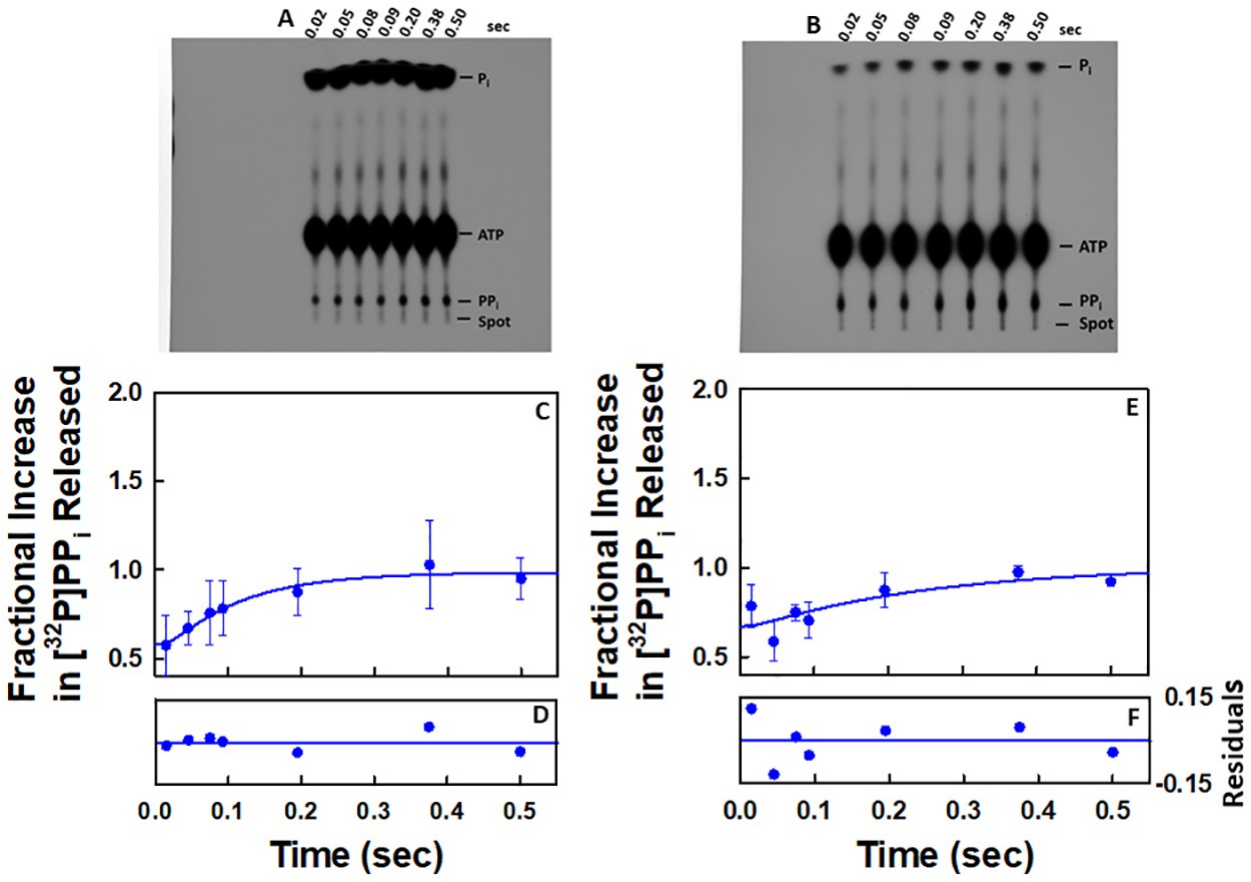
QF-TLC results for pyrophosphate release in the presence of [γ-^32^P]ATP and UTP as well as [γ-^32^P]ATP and GTP, respectively, during one round of the NAC. The autoradiograms correspond to the time-dependent release of [^32^P]PP_i_ in the presence of (**A**) 0.5 μM EC, 50 μM [γ-^32^P]ATP [60 μCi/pmol] and 50 μM UTP at 25°C and (**B**) 0.5 μM EC with 50 μM [γ-^32^P]ATP [60 μCi/pmol] and 50 μM GTP, respectively, after mixing at 25°C. Averages for each data set in C and E were obtained as indicated in Methods. The non-zero y-intercept in each case is due to contaminating [^32^P]PP_i_ in the reaction mixtures. (**C**) Plot of the time dependent release of [^32^P]PP_i_ in the presence of 0.5 μM EC, 50 μM UTP and 50 μM [γ-^32^P]ATP [60 μCi/pmol] after mixing. The data correspond to the average of three independent experiments. The line through the data points was generated by fitting the data to the model in Scheme 5. Plot of time dependent release of [^32^P]PP_i_ in the presence of 0.5 μM EC, 50 μM GTP and 50 μM [γ-^32^P]ATP [60 μCi/pmol] after mixing at 25°C (E). The data correspond to the average of two independent experiments. The line through the data points was generated by fitting the data to the model in Scheme 5.

Representative autoradiograms obtained for the time dependencies of pyrophosphate release in the presence of ATP and either UTP or GTP as monitored by QF-TLC are given in Fig 10A and 10B, respectively. In the case of UTP, the experiment was conducted three times. The autoradiograms of the other two experiments are given in S1 Raw images along with a larger version of the image in Fig 10A. In the case of GTP, the experiment was conducted twice. The autoradiogram of the other experiment is given in S1 Raw images. Because the fractional increases of pyrophosphate release in the cases of the noncognate nucleotides (GTP and UTP) shown in Fig 10C and 10E are both comparable to the result obtained in the presence of [γ-^32^P]ATP alone, Scheme 5 was used to analyze the data. Because of the limited number of data points obtained in each QF-TLC experiment, it was not possible to optimize the values for more than one parameter. Only the value of *k*_*off*,*PP*_ was optimized in the fits. In the modeling of the data by using Scheme 5, the value of the rate constants for pyrophosphate release in the presence of UTP is 10 s^-1^ (Table 9) and in the presence of GTP it is 4 s^-1^ (Table 9). There is good agreement between the data points and the proposed model in Scheme 5 (Fig 10C and 10E) as indicated by the residuals (Fig 10D and 10F) and the constraints of the values of *k*_*off*,*PP*_ (Table 9). This further establishes that only the cognate NTP for incorporation increases the rate of release of the pyrophosphate generated by incorporation of the previous nucleotide.

**Table 9:** Values of rate constants for *E*. *coli* core RNA polymerase during one round of the NAC in the presence of [γ-^32^P]ATP and the noncognate nucleotides as monitored by QF-TLC.

	Parameter	Value	Lower Bound	Upper Bound
	*k*_*1*_ *(i*.*e*., *k*_*on*,*ATP*_*)*	100 μM^-1^ s^-1^	--	--
	*k*_*2*_ *(i*.*e*., *k*_*translocation*_*)*	137 s^-1^	--	--
	*k*_*3*_ *(i*.*e*., *k*_*AMP incorporation*_*)*	70 s^-1^	--	--
Noncognate NTP				
UTP	*k*_*4*_ *(i*.*e*., *k*_*off*,*PP*_*)*	10 s^-1^	8 s^-1^	12 s^-1^
GTP	*k*_*4*_ *(i*.*e*., *k*_*off*,*PP*_*)*	4 s^-1^	1 s^-1^	7 s^-1^

The observable output expression is defined as (a*PP_i_ + b) in the fitting routine where only free pyrophosphate is monitored. The estimate for *k*_*translocation*_ is the average of the four values for this rate constant given in Tables 1, 5 and 6 and the estimate for *k*_*AMP incorporation*_ is approximated by the average of the four values of *k*_*UMP incorporation*_ given in Tables 1, 5 and 6. Only *k*_*4*_ was optimized in the fitting routine.

## Discussion

The interplay between nucleotide incorporation and pyrophosphate release of *E*. *coli* RNA polymerase during transcription was investigated in rapid kinetic studies during one and two rounds of the NAC. Seven major findings from this study were considered in formulating a mechanism for the NAC during processive transcription.

1. **Pyrophosphate release from RNA polymerase elongation complexes during a single round of the NAC is slow relative to the rate of nucleotide incorporation**. The stopped flow kinetic data for pyrophosphate release during one round of the NAC are consistent with a minimal model involving four steps (Scheme 1). The first step is the binding of the nucleotide to the pre-translocated state of the EC. This is followed by translocation of the enzyme along the DNA template to form the post-translocated state in the second step. The third step involves the incorporation of the nucleotide and the generation of pyrophosphate. In the fourth step, the pyrophosphate is released. The rate limiting step for one round of the NAC is the release of pyrophosphate (Table 1). The value of the rate constant for pyrophosphate release during one round of the NAC as monitored in stopped flow kinetic studies (10 s^-1^; Table 1) is comparable to those reported previously in pyrophosphate binding studies (5.7±1.6 s^-1^ to 8.1±4.8 s^-1^) [37]. These values of the rate constants are significantly less than those reported in a previous study (77–115 s^-1^) [41]. This is addressed in more detail in section 4 below. The analysis of one round of the NAC by monitoring pyrophosphate release in the current stopped flow kinetic studies also provided an estimate for the rate constant corresponding to the translocation of the core polymerase from the pre- to the post-translocated state. The values of the rate constant for this translocation varied from 70–157 s^-1^ (Tables 1 and 6). These values are consistent with the ones reported in a previous study (59–96 s^-1^; S1 Table [41]). The value of the rate constant for pyrophosphate release is approximately 7 times less than that for nucleotide incorporation (10 s^-1^ versus 68 s^-1^; Table 1).
2. **The stopped flow kinetic data set for pyrophosphate release is not consistent with a model in which the slow release of pyrophosphate is due to a slow rate of nucleotide incorporation during one round of the NAC**. To test the hypothesis that the slow rate of pyrophosphate release may be due to a slow rate of nucleotide addition under conditions of a final nucleotide concentration of 50 μM in the current study, the value of the rate constant for nucleotide incorporation was set constant at 10 s^-1^ and KinTek Explorer was used to determine if this would force the value of the rate constant for pyrophosphate release to be 10 s^-1^ according to the model given in Scheme 1. The poor constraints on the fitted values of the rate constants for translocation and pyrophosphate release (Table 2) rule this out as a viable mechanism based on the stopped flow kinetic data set for pyrophosphate release. The rate limiting step for the NAC according to Scheme 1 is the release of pyrophosphate and not nucleotide incorporation.
3. **The stopped flow kinetic data set for PP**_**i**_ **release is not consistent with a model for one round of the NAC in which nucleotide binding occurs after translocation of the core polymerase from the pre- to the post-translocated state**. The prevailing model for the NAC involves the oscillation of the enzyme between the pre- and post-translocated state [11–18]. Entry of nucleotides into the active site is thought to trap the enzyme in the post-translocated state thereby allowing the nucleotides to be incorporated. The classical linear ratchet mechanism outlined above posited that the nucleotides enter the active site through the secondary channel which is open only in the post-translocated state [12, 48–50]. This classical thermal ratchet mechanism was modified over time to accommodate a mechanism for the binding of incoming nucleotides to a secondary nucleotide binding site when the core polymerase is in the pre-translocated state with the secondary channel closed [14, 34, 51]. There was no mention of the location of the secondary nucleotide binding site nor the channel that the nucleotides use to enter the site. In our current studies, the failure to obtain a constrained value for the rate constant corresponding to translocation of the core polymerase from the pre- to the post-translocated prior to nucleotide binding (Table 3) is also consistent with the nucleotide binding to the core polymerase in the pre-translocated state. Later in the discussion, we propose a nucleotide driven mechanism for the NAC based on the incoming nucleotide binding to the core polymerase in the pre-translocated state.
4. **Embedded in data reporting to show rapid pyrophosphate release from RNA polymerase after CMP incorporation (*k***_***off*,*PP***_ **= 104 s**^**-1**^**) is a region that is consistent with slow pyrophosphate release (*k***_***off*,*PP***_ **= 4 s**^**-1**^**) during a single round of the NAC**. The prevailing view is that pyrophosphate release after nucleotide addition is rapid relative to nucleotide incorporation. Previous studies on pyrophosphate release appeared to support this hypothesis [41]. However, a reinvestigation of those studies reveals inconsistencies. Embedded in the pyrophosphate release curve (Fig S3A [41]) is a region that is consistent with a slower rate of pyrophosphate release (*k*_*off*,*PP*_ = 4 s^-1^, Table 4). This is comparable to the values obtained in the current study (*k*_*off*,*PP*_ = 10 and 8 s^-1^, Tables 1 and 6) for pyrophosphate release during one round of the NAC. This establishes that the slow rate of pyrophosphate release in our studies (*k*_*off*,*PP*_ = 10 and 8 s^-1^, Tables 1 and 6) is not an artifact due to different experimental conditions. It was also demonstrated in our studies that the perturbation in the region between 0.08 and 0.7 seconds is due to the reaction catalyzed by RNA polymerase. This was accomplished by using a functional assay which indicated that the pyrophosphate equivalents released over this time range were proportional to the number of nucleotides incorporated.
  In the previous study [40], the lack of the use of pyrophosphate and phosphate mops in all the reaction mixtures raises serious problems in the interpretation of data. The presence of pyrophosphate and/or phosphate contaminants in the reaction mixtures would lead to a rapid increase in fluorescence with values of the rate constant equal to or greater than 100 s^-1^. This increase in fluorescence could be mistaken for the release of pyrophosphate during the NAC. If the release of pyrophosphate during the NAC proceeds at a more moderate rate, there would be several time regions displaying an increase in fluorescence. This would make it difficult to discern which time region corresponds to pyrophosphate release during the NAC.
5. **The presence of the next cognate nucleotide for incorporation increases the rate of release of pyrophosphate generated from incorporation of the preceding nucleotide; noncognate nucleotides have no effect on pyrophosphate release**. The stopped flow kinetic data set for pyrophosphate release during two rounds of the NAC is consistent with a minimal model involving six steps (Scheme 4). The first step is the binding of the first nucleotide for incorporation to the pre-translocated state of the EC. This is followed by translocation of the enzyme along the DNA template to form the post-translocated state accompanied by entry of the nucleotide into the active site in the second step. The third step involves the incorporation of the nucleotide and the generation of the first pyrophosphate equivalent. In the fourth step, the second nucleotide for incorporation binds to the EC.PP_i_ complex in the pre-translocated state. The fifth step is treated as a composite of three processes referred to as the triad that involves release of the first pyrophosphate equivalent, translocation of the core polymerase to the post-translocated state and incorporation of the second nucleotide. Based on the relative values of the various rate constants for one round of the NAC (Table 1), the rate limiting process in the triad is the release of pyrophosphate. As such, the rate of pyrophosphate release limits the rates of translocation and nucleotide incorporation. The net result is that all three processes in the triad proceed with the same rate constant. The sixth step is the release of the second pyrophosphate equivalent.
  When bound to the EC, the first pyrophosphate equivalent reduces the value of the rate constant for translocation by a factor of approximately 11 (197 s^-1^ versus 18 s^-1^; Table 5). It also reduces the value of the rate constant for nucleotide incorporation by a factor of approximately 4 (68 s^-1^ versus 18 s^-1^; Table 5). The value of the rate constant for the release of the second pyrophosphate equivalent is 6 times less than that for the first pyrophosphate equivalent (18 s^-1^ versus 3 s^-1^; Table 5).
  The binding of the next cognate nucleotide for incorporation to the EC.PP_i_ complex increases the value of the rate constant for pyrophosphate release by a factor of approximately 2 (10 s^-1^; Table 1 versus 18 s^-1^; Table 5). This is not sufficient to increase the rate of incorporation of the second nucleotide to a value comparable to that observed for incorporation of the first nucleotide (18 s^-1^ versus 68 s^-1^; Table 5).
  Similar decreases in the rate constants for incorporations of subsequent nucleotides after incorporation of the first one were reported previously in quench-flow kinetic studies conducted with both *E*. *coli* and eukaryotic RNA polymerases [45] as well as with HIV reverse transcriptase when using RNA as a template in a combination of quench-flow and stopped-flow kinetic studies [52]. In both studies, the results were interpreted to indicate that pyrophosphate release is the rate limiting step during processive transcription [45, 52]. In the case of *Staphylococcus aureus* replicative polymerase PolC, the rate of pyrophosphate release is slow relative to nucleotide incorporation during one round of the NAC [53]. However, unlike in the case of RNA polymerase, the binding of subsequent nucleotides for incorporation eliminates the slow release of pyrophosphate.
  In single molecule studies with RNA polymerase, it was also concluded that pyrophosphate release is the rate limiting step during processive transcription [54].
6. **Quench flow-TLC (QF-TLC) studies support the stopped-flow kinetic results indicating that pyrophosphate release after one round of the NAC is slow relative to nucleotide incorporation and that the second nucleotide for incorporation increases the rate of release of the first pyrophosphate equivalent**. Although the number of data points collected in the QF-TLC studies is not sufficient to obtain robust fitting that leads to values for each of the respective rate constants, these studies allow one to test the postulated model(s) derived from the stopped flow kinetic studies. Due to the nature of the assay in which [γ-^32^P]ATP is used to monitor pyrophosphate release during one and two rounds of the NAC, one can use the formation of ^32^PP to track the release of the first pyrophosphate equivalent without interference from the release of the second one during two rounds of the NAC. This allows one to directly compare the raw data obtained from one and two rounds of the NAC. The raw data (*i*.*e*., the data points alone, Fig 9C) obtained in the QF-TLC studies indicate that the release of the first pyrophosphate equivalent during two rounds of the NAC is greater than the release of pyrophosphate during one round of the NAC. This supports the hypothesis that the next nucleotide for incorporation increases the rate of release of the first pyrophosphate equivalent. By using KinTek to simulate pyrophosphate release curves and optimizing only for the value of *k*_*off*,*PP*_, one observes that pyrophosphate release and not nucleotide incorporation or translocation is the rate limiting step during one round of the NAC (Table 7). One also observes that although the next cognate nucleotide for incorporation increases the rate of release of the first pyrophosphate equivalent during two rounds of the NAC, the rate of incorporation of the second nucleotide is slower than what one would expect based on the rate of nucleotide incorporation during one round of the NAC (Tables 7 and 8).
7. **QF-TLC studies support the stopped-flow kinetic results indicating that only the cognate nucleotide for incorporation increases the rate of release of the first pyrophosphate equivalent**. Once again, robust fitting of the datasets obtained in QF-TLC studies to multistep models leading to estimates of the rate constants for all the parameters is not possible because of too few data points. However, the raw data indicate that noncognate nucleotides do not elicit an increase in the release of the first pyrophosphate equivalent. This indicates that interactions between the cognate nucleotide for incorporation and the template DNA strand are crucial to elicit an increase in the rate of pyrophosphate release.

The results from the stopped flow and QF-TLC kinetic studies indicate that the rate of pyrophosphate release after nucleotide addition is slow in the absence of the next nucleotide for incorporation (*k*_*off*,*PP*_ = 3–10 s^-1^); but it increases when the next cognate nucleotide for incorporation is present (*k*_*off*,*PP*_ = 18–28 s^-1^). In a previous stopped flow kinetic study [40], the rate of release of pyrophosphate after nucleotide addition was also found to be slow in the absence of the next nucleotide for incorporation (*k*_*off*,*pyrophosphate*_ = 3 s^-1^). However, the reported increase in the rate of pyrophosphate release in the presence of the next nucleotide for incorporation was significantly greater (*k*_*off*,*pyrophosphate*_ = 500 s^-1^). In the previous study, the stopped flow kinetic studies were based on a different coupled enzyme assay involving changes in absorbance rather than fluorescence. The amplitudes of the changes were small (0.0000 to 0.0020 absorbance units) and there was significant scatter in the data. Data analyses were conducted on the pyrophosphate release curves by single exponential fitting and not by modeling of the reaction. All these factors most likely contributed to the erroneously high value for the release of pyrophosphate in the presence of the next cognate nucleotide for incorporation. This value is inconsistent with the current study which is based on a more robust coupled enzyme assay to monitor pyrophosphate release in stopped flow kinetic studies as monitored by changes in fluorescence. Also, the QF-TLC kinetic studies provide a system to monitor release of the first pyrophosphate equivalent during two rounds of the NAC without interference from the release of the second pyrophosphate equivalent.

Collectively, the results from rapid kinetic studies on one and two rounds of the NAC are consistent with an NTP-driven mechanism for transcription. With the core polymerase in the pre-translocated state after nucleotide incorporation, the next nucleotide for incorporation most likely binds to its complementary base located in the downstream DNA of the elongation complex prior to pyrophosphate release. This could then lead to conformational changes in the elongation complex that trigger a cascade of events leading to (a) the more rapid release of the pyrophosphate generated from the previously incorporated nucleotide, (b) translocation of the core polymerase from the pre- to the post-translocated state accompanied by the entry of the pre-selected nucleotide into the active site followed by (c) incorporation of the nucleotide. In the modelling of the steps during two rounds of the NAC as monitored by pyrophosphate release, a composite rate constant composed of these three processes was postulated. Conformational changes induced by the binding of the next nucleotide for incorporation downstream from the active site would have to be synergistic in nature leading to alterations at the active site as well as a downstream nucleotide binding site (the CH3 pocket) that result in triggering the triad of processes.

**Fig 11:**
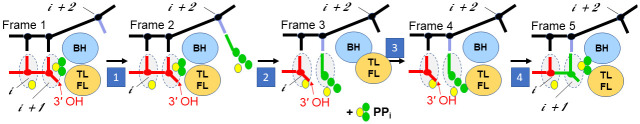
An NTP-driven mechanism for the nucleotide addition cycle of Escherichia coli RNA polymerase during transcription. Template DNA strand is shown in black, RNA strand in red, incoming NTP along with PP_i_ in green and Mg^2+^ in yellow. BH is the bridge helix which separates the active site from the downstream DNA, TL is the trigger loop and FL is the fork loop. The active site corresponds to *i + 1* and the position one nucleotide downstream from the active site corresponds to *i + 2*.

The results from the rapid kinetic studies must be reconciled with the structure and properties of RNA polymerase. Immediately after nucleotide incorporation, the core polymerase is in the pre-translocated state with the secondary channel closed (Fig 11). The closure of the secondary channel is mediated through the action of the trigger loop and the F-loop. The pyrophosphate is trapped at the active site which aligns with the *i + 1* register on the DNA. To trigger a conformational change in the elongation complex, the nucleotide for incorporation would have to bind to its complementary base at the *i + 2* register on the template strand. With the secondary channel closed, a different pathway for entry into the *i + 2* site would have to be utilized by the next nucleotide for incorporation. The cognate nucleotide may interact with the *i + 2* register by entering the CH3 pocket through one of the CH3A/B subchannels. The CH3A/B along with the CH3C/D subchannels were first identified in aMD simulation studies on human RNA polymerase II in the presence of the TFIIF transcription factor [29, 30]. By inspection [30], comparable CH3A/B but not the CH3C/D subchannels were found to be present in bacterial RNA polymerase (PDB#4LYN, 4YLO AND 4YLP) [31] as well as in archaeal bacteria (PDB#4V8S) [55]. The CH3 pocket is located within the CH1 (channel 1) macro-channel that surrounds the downstream DNA helical duplex and downstream template strand. Further aMD simulation studies indicated that the occupancy of the *i + 2* site by the cognate nucleotide for incorporation leads to synergistic coupling of conformational changes between the downstream nucleotide binding site (CH3P) and the active site. This provides a mechanism by which the binding of the next nucleotide for incorporation could trigger the more rapid release of pyrophosphate at the active site. In additional aMD simulation studies, it was illustrated how nucleotides could be transferred from the CH3 pocket to the active (*i + 1*) site. The bridge helix separates the active (*i + 1*) site from the downstream DNA which begins at the *i + 2* register. In the aMD simulation of the transfer of NTPs into the active site, the bridged helix along with other functional domains that include the trigger loop, fork loop 2, the F loop and the F claw were shown to undergo synergistic conformational changes.

### Proposed model for the NAC of RNA polymerase during processive transcription

An NTP-driven model for the NAC during processive transcription is consistent with the rapid kinetic results obtained in this study and the information provided by aMD simulation studies [29, 30] (Fig 11). Frame 1 corresponds to the elongation complex just after incorporation of a nucleotide. The core polymerase is in the pre-translocated state with pyrophosphate trapped at the active site by the fork loop and the F loop that gate the secondary channel (CH2). In step 1, the cognate nucleotide for incorporation is postulated to enter the CH3 pocket by way of one of the CH3A/B subchannels and interacts with its complementary NMP (nucleoside monophosphate) in the template strand. This could then trigger a synergistic conformational change between the CH3 pocket and the active site [30] in step 2 that would then allow pyrophosphate to exit through the opened secondary (CH2) channel as the core polymerase undergoes translocation from the pre- to the post-translocated state. The translocation of the core polymerase in step 2 would then allow the next nucleotide for incorporation to transit into the active site along with its complementary NMP in the template strand. According to the aMD simulation studies [29, 30], the next nucleotide for incorporation along with its complementary NMP enter the active site separately and then recombine in the active site. The active site would then close as shown in step 3, thereby positioning the nucleotide for chemistry as shown in step 4. This step would then return the cycle to the state with the pyrophosphate trapped at the active site. In the mechanism given in Fig 11, steps 2 through 4 correspond to the triad of processes proposed in the model in Scheme 4.

Although the Brownian-ratchet model is the prevailing model for the NAC of RNA polymerase, the NTP-driven model provides an alternative model for the NAC that explains the rapid kinetic results reported in this study for multiple rounds of transcription. Based on the results from the current study, the mechanisms for pyrophosphate release during multiple rounds and a single round of transcription are different. The more rapid release of pyrophosphate during multiple rounds of transcription appears to be due to an NTP-driven mechanism whereas the release of pyrophosphate during a single round of transcription may be due to Brownian motion of the core polymerase that allows pyrophosphate to escape through the secondary channel when the enzyme is in the post-translocated state. A Brownian motion mechanism for pyrophosphate release may occur at termination sites and perhaps at RNA polymerase pause sites. The mechanisms for nucleotide addition are also different during multiple and single rounds of transcription. The rate of incorporation of the next nucleotide may be limited by the release of pyrophosphate from the previously incorporated nucleotide during multiple rounds of transcription whereas more rapid nucleotide incorporation is observed during a single round of transcription because it is not limited by pyrophosphate release. Within the cell, more rapid nucleotide incorporation may occur during formation of the first phosphodiester bond in the RNA product and may also occur when RNA polymerase is released from pause sites [56, 57] or after it escapes from transcriptional arrest [58].

An NTP-driven model for the NAC is analogous to a power stroke model in which conformational changes in the protein are used to do mechanical work [59]. In the case of the NAC, the NTP-driven model appears to involve the more rapid release of pyrophosphate, the translocation of the core polymerase from the pre- to the post-translocated state accompanied by the entry of the pre-selected nucleotide into the active site from the CH3 pocket. Because the aMD simulations indicate that the binding of the next cognate nucleotide for incorporation in the CH3 pocket elicits synergistic conformational changes in the elongation complex, it appears that it is the nucleotide binding step rather than the release of pyrophosphate that initiates the power stroke. Also, the robust incorporation of nucleotides during one round of the NAC supports an NTP-driven mechanism rather than one based on pyrophosphate release.

Power stroke and Brownian ratchet mechanisms are not mutually exclusive. They have been demonstrated to be used by the same protein to varying degrees to perform biological functions [60, 61]. An interplay between power stroke and Brownian ratchet mechanisms may be required for RNA polymerase to carry out all its tasks during transcription. However, power stroke mechanisms generally “outperform” Brownian ratchet mechanisms in terms of speed, power and efficiency [62].

## Materials and methods

All RNA and DNA oligonucleotides were purchased from Integrated DNA Technologies. [γ-^32^P]ATP was obtained from PerkinElmer Health Sciences, Inc. HiTrap^™^ Heparin HP columns (1 ml bed volume) were purchased from GE Healthcare. TLC PEI Cellulose F sheets were obtained through Fisher Scientific. All other reagents were of the highest purity available from commercial sources.

### Growth and purification of A197C phosphate binding protein (PBP)

The ANCC75 bacterial strain containing plasmid pSN5182 that encodes the A197C mutant of the *E*. *coli* phosphate binding protein (PBP) was obtained from Dr. Martin R. Webb. Bacterial growth was carried out as follows. **(1)** Transformed competent BL21 cells containing the mutated A197C-PBP gene on the plasmid were grown up overnight on an LB agar plate containing 100 μg/mL of ampicillin. **(2)** Inoculate each of two 50-mL starter cultures containing 100 μg/mL ampicillin in LB broth with a colony from the LB agar plate and incubate overnight with vigorous shaking at 37°C. **(3)** Start four 500 mL LB + 100 μg/mL ampicillin growths by using a ~1/50 dilution of the overnight growth; *i*.*e*., 10 mL of culture per 500 L in four 2-liter (baffled) flasks. Incubate at 37°C with vigorous shaking until the A600nm is 1. **(4)** Transfer cells to 22°C; after ~1 hour, add IPTG to 1000 μM (*i*.*e*., 5 mL of 100 mM stock IPTG per 500 mL) and grow overnight (~17 hours) at 22°C. **(5)** Harvest the cells by centrifugation at 4000 rpm for 25 min at 4°C in a GS-3 rotor. **(6)** Resuspend the pellets in a total of 350 mL of 20 mM Tris-HCl pH 8.0, 1 mM EDTA, 5 mM β-ME. **(7)** Pool the suspensions into one centrifuge bottle and subject to centrifugation at 4000 rpm for 25 min at 4°C in a GS-3 rotor. Store pellet at -80°C. **Cell disruption was carried out as follows. (1)** Thaw pellet and resuspend in 35 mL of 20 mM Tris-HCl pH 8.0, 1 mM EDTA, 5 mM β-ME. **(2)** Subject cells to sonication. Sonicate in aliquots of no more than 40 mL on ice for 10 sec intervals followed by no sonication for 10 sec. Repeat cycle for a total of 2 min per aliquot. Ensure a homogeneous mixture. **(3)** Combine aliquots and pellet cell debris by centrifugation in a SS34 rotor at 11,000 rpm for 45 min at 4°C. **(4)** Collect the clear supernatant in a graduated cylinder (50 mL); an aliquot (100 μL) is collected and labeled as crude extract for later electrophoretic analysis. Discard the precipitate. **(5)** Load supernatant onto a 120 mL Q Sepharose FF column, equilibrated in 10 mM Tris-HCl pH 7.6, 1 mM β-ME. Wash with two column volumes in the same buffer with a flow rate of 1 mL/min. Apply a one-liter gradient of 0–200 mM NaCl in this buffer. Monitor at 280 nm. **(6)** Pool PBP and concentrate to 1–1.5 mM (*i*.*e*., 37–55 mg/mL) by using an Amicon Ultra Centrifugal filter (10,000 molecular weight cut-off). Final volume should be between 4 & 5 mL. Perform a BCA analysis prior to concentrating to determine how much the sample must be reduced in volume. **(7)** Dialyze sample against storage buffer (10 mM Tris-HCl pH 7.6, 10 mM MgCL_2_, 0.1 M NaCl, 0.1 mM DTT and 50% glycerol) and store at -20°C. (Make certain that the cut-off limit of the dialysis tubing is appropriate.) Do a BCA assay.

#### Labelling of A197C PBP with MDCC 7-Diethylamino-3-[N-(2-maleimidoethyl)carbamoyl]-coumarin

Labeling was carried out as described previously [63] with recommended modifications [64].

### Purification of his-tagged *E*. *coli* pyrophosphatase

#### Bacterial growth for pET47b EPPA

**(1)** From frozen stock, streak bacteria containing plasmid on a Kanamycin plate. Incubate overnight at 37°C. **(2)** Start two 50 mL LB + 50 μg/mL antibiotic (Kanamycin; Kn) growths from a single colony in 125 mL Erlenmeyer flask; incubate at 37°C overnight at 225 rpm. **(3)** Start four 750 mL LB + 50 μg/mL antibiotic (Kn) growths by using ~1/50 dilution of the overnight growth; *i*.*e*., 15 mL of culture per 750 mL in 2-liter Erlenmeyer flasks. **(4)** Incubate at 37°C with shaking at 225 rpm until the OD600 is at or over 0.4 (*i*.*e*., 2–4 hrs.). **(5)** Transfer flasks to 22°C; after ~1 hour, add IPTG to a final concentration of 0.5 mM; *i*.*e*., 5 mL of 100 mM stock IPTG to 1000 mL. **(6)** Grow overnight (~17 hours) at 22°C w/shaking. **(7)** Harvest cells by centrifugation in a GS-3 rotor at 6000 rpm for 10 minutes. **(8)** Suspend pellet in 3 mL of buffer A per gram of cell paste (*e*.*g*., 35 mL) before freezing at -80°C Buffer A: 20 mM Tris-HCl pH 8.0, 5 mM MgCL_2_, 0.5 M NaCl, 40 mM imidazole and 1 tablet of protease inhibitor cocktail per 10 mL. **Cell disruption is carried as follows: (1)** Thaw frozen cell pellet on ice and add protease inhibitor (1 tablet of protease inhibitor cocktail per 10 mL). Use orbital rotator in cold box to mix. **(2)** Incubate cells at 4°C with gentle agitation for 20 min. **(3)** Sonicate in aliquots of no more than 40 mL on ice for 10 sec intervals followed by no sonication for 10 sec. Repeat cycle for a total of 2 min per aliquot. (Ensure a homogeneous mixture.) **(4)** Combine aliquots and pellet cell debris by centrifugation in a SS34 rotor at 12–14,000 rpm for 45 min at 4°C. **(5)** Collect the clear supernatant in a graduated cylinder. Discard the precipitate. **Ni-agarose chromatography as follows: (1)** Equilibrate column with Ni-column loading buffer, Buffer A (20 mM Tris-HCl pH 8.0, 5 mM MgCL_2_, 0.5 M NaCl, 40 mM imidazole and 1 tablet of protease inhibitor cocktail per 10 mL). **(2)** Load clear supernatant onto Ni-agarose column (5–7 mL bed volume) until absorbance returns to baseline. **(3)** Wash column with 60 mL buffer B (20 mM Tris-HCl pH 8.0, 5 mM MgCL_2_, 0.15 M NaCl, 40 mM imidazole) until absorbance returns to baseline. **(4)** Elute pyrophosphatase with elution buffer; 30 mL of Buffer C (20 mM Tris-HCl pH 8.0, 5 mM MgCL2, 0.15 M NaCl, 500 mM imidazole). **(5)** SDS-Page gels to confirm fractions, for dialysis. **(6)** Pool fractions. Then use Nanodrop A280 to find protein concentration. (Want a concentration of ~18–20 μM final. **(7)** Dialyze sample against dialysis buffer I (50 mM Tris-HCl pH 8.0, 5 mM MgCL2, 0.15 M NaCl, 0.1 mM DTT and 5% glycerol) to remove imidazole. 2 times overnight both times. **(8)** Dialyze sample against final storage (10 mM Tris-HCl pH 8.0, 5 mM MgCL2, 0.15 M NaCl, 0.1 mM DTT and 5% glycerol) and store at -20°C.

### Assembly of elongation complexes (EC)

The method of Komissarova *et al*. [65] as modified by Johnson *et*. *al*. [40] was used for assembling elongation complexes. The Pierce^™^ BCA (bicinchoninic acid) assay kit was used to determine the concentration of the elongation complexes according to the Pierce Chemical Co. protocol. Because purification of the elongation complexes after assembly as indicated in the protocol given in Johnson *et al*. [40], there was no free nucleic acids present in the system.

### Stopped-flow kinetic studies

Pyrophosphate release after nucleotide addition was measured in stopped flow kinetic experiments by using a coupled enzyme assay under various conditions in HEPES buffer (10 mM HEPES; pH 8.0, 10 mM MgCl_2_, 0.05 M KCl and 0.1 mM DTT) at 25°C. The general approach was first developed by Hanes & Johnson [66] based on studies by Brune *et al*. [67]. In the assays, pyrophosphatase (PPase) converts pyrophosphate to phosphate and then the phosphate binds to the fluorescently labelled phosphate binding protein (MDCC-PBP). Rapid kinetic measurements of the alterations in fluorescence were performed by using an Applied Photophysics SX20 stopped-flow spectrometer. The excitation wavelength was 430 nm and fluorescence from the samples was monitored after passing through a 455-nm cut-off filter (Oriel). The protocol for conducting these experiments involved mixing equal volumes of two solutions each of which has been treated with both a phosphate and a pyrophosphate mop. The elongation complex solutions contained 2 μM PPase, 0.2 mM 7-methylguanosine (MEG) and 0.1 IU/mL of purine nucleoside phosphorylase (PNP) along with the appropriate amounts of EC, whereas the nucleotide solutions contained 100 picomolar PPase, 0.2 mM MEG, 0.1 IU/mL of PNP along with 10 μM MDCC-PBP and the appropriate amounts of nucleotides. All the above correspond to pre-mixing concentrations. These solutions were preincubated separately for 15 minutes to reduce phosphate and pyrophosphate contamination. In the case of the EC solutions, the MEG and PNP combination serves as the phosphate mop whereas the MEG, PNP and PPase (2 μM) combination serves as the pyrophosphate mop. In the case of the nucleotide solutions, the MEG and PNP combination serves as the phosphate mop whereas the MEG, PNP and PPase (100 picoM) combination serves as the pyrophosphate mop. All the above correspond to pre-mixing concentrations. The syringes and lines in the stopped-flow apparatus were subjected to a phosphate mop containing 0.2 mM MEG, and 1.0 IU/mL of PNP in HEPES buffer for 10 minutes. Appropriate controls were run in the absence of elongation complexes to determine the background variation in fluorescence over time. In each case, the background was subtracted from the results obtained in the presence of the elongation complexes. In the case of the stopped flow kinetic studies with UTP as well as with UTP and ATP, respectively, in the presence of the elongation complex, a split time base of 0 to 0.05 sec and 0.05 to 0.5 sec was used in the collection of the data. This was done to determine if ATP triggered an exceptionally large increase in the rate of release of the first pyrophosphate equivalent within the first-time base. When an exceptionally large increase was not observed, a single time base was used over the range of 0 to 0.5 seconds to ascertain the effects of the noncognate nucleotides (GTP and CTP) in the presence of UTP and the elongation complex. All the stopped flow kinetic experiments were not conducted with the same EC preparation and each experiment corresponds to repeated measurements of the same sample.

### Determination of pyrophosphate equivalents released in the NAC as monitored in stopped flow kinetic experiments

The rationale behind this approach is that the ratio of the endpoints for the release of pyrophosphate by the reaction catalyzed by the elongation complexes during two rounds and one round of the NAC should be two. For this purpose, the settings on the stopped flow apparatus including the detector voltage were kept reasonably constant for the series of experiments performed in this study. The relative fluorescence of each data set was extrapolated to its endpoint at approximately 2 seconds. There were four equations generated in this case. For the cognate nucleotides for incorporation (UTP and ATP), the equation was 0.87 = 2x; for UTP only, it was 0.25 = x; for UTP and the noncognate GTP, it was 0.37 = x and for UTP and noncognate CTP, it was 0.29 = x, where x corresponds to the value of relative fluorescence units that correspond to one pyrophosphate equivalent. Summing the equations and solving for x led to a value of 0.36 relative fluorescence units corresponding to approximately one pyrophosphate equivalent. Each plot for the pyrophosphate release curve contains the relative fluorescence on the left y-axis and the pyrophosphate equivalents on the right y-axis of the graph. The data are internally consistent. The value of the ratio of pyrophosphate equivalents released for two rounds of the NAC versus one round is approximately two.

### Extraction of data points from a published pyrophosphate release curve

After expansion of the captured screenshot of the published pyrophosphate curve during one round of the NAC [41], vertical lines were extended from the x-axis to the pyrophosphate release curve. Next horizontal lines were extended to the y-axis from the intersection of the vertical lines with the pyrophosphate release curve. It was found that 14 mm corresponded to approximately 0.125 a.u. (arbitrary units) on the y-axis from the expanded plot. Therefore, 0.125 a.u./14 mm = 0.0089 a.u./1 mm. A new zero was assigned to the 0.75 value from the original plot. Values in a.u. for six time points were determined by multiplying the mm value of each point from the new zero point by 0.0089 a.u./1 mm. After fitting the data by using KinTek Explorer, the data was normalized by extrapolating the curve generated in the fitting to 5 seconds. The value of the fluorescence at 5 sec was then used to normalize the data to obtain the relative fluorescence change as a function of time.

### Quench-flow thin Layer Chromatography (QF-TLC) kinetic studies

To monitor pyrophosphate release as a function of time in quench-flow experiments requires a quenching agent that does not interfere with this process. Both acid (HCl) quenching and pulse-chase would interfere with the pyrophosphate release. Quench-flow experiments were conducted by using a Kin Tek computer-controlled chemical-quench-flow apparatus (Model RQF-3) as described previously [40]. The reaction buffer was HEPES buffer (10 mM HEPES (pH 8.0), 10 mM MgCl_2_, 0.05 M KCl and 0.1 mM DTT) and experiments were carried out at 25°C. The samples were resolved by TLC with a KH_2_PO_4_ buffer (0.3 M; pH 7.0). Sample sizes loaded on the TLC (PEI Cellulose F sheets) were 1 μL. The TLC sheets on which the samples were run were subjected to autoradiography and the resulting autoradiograms were analyzed by using Un-Scan-It. The final figures for the manuscript were generated with Bio-Rad’s ChemiDoc. **Averaging of data sets was carried out as follows**. Each data set was normalized prior to averaging. Normalization involved extrapolating each data set to 2 minutes to obtain the endpoint. The value of the intensity of each point was then divided by the value at the endpoint. At this point, the data sets were averaged.

### Kinetic simulations and data plotting

Kinetic simulations were performed by using Kin Tek Global Explorer Professional Version 6.3 (Kin Tek Corp., Austin TX) [32]. The procedure involves importing the data into the program and entering a model to test along with the initial values for the concentrations of the substrate(s) and elongation complexes. For each simulation, the residuals (yobserved–y_calculated_) were determined as an aid in evaluating the overall goodness of the fit to the postulated model. The other factor that is important for evaluating the overall goodness of the fit is how well the values of the parameters are constrained. This was accomplished by determining the constraint limits of each parameter that was allowed to float during the fitting [33]. This latter condition refers to the uncertainties in the values of the parameters as determined by the program. If both conditions are not met, then the model is not a good representation of the mechanism of action of the enzyme. Plots of the data were generated by using SigmaPlot 11.2 (Systat Software Inc).

## Supporting information

S1 Raw images (PDF)
