# Supplementary material for: An NTP-driven mechanism for the nucleotide addition cycle of Escherichia coli RNA polymerase during transcription
Source: PLoS One. 2022 Oct 25;17(10):e0273746. doi: 10.1371/journal.pone.0273746 (PMC9595533; doi:10.1371/journal.pone.0273746)
Supplement: S1 Raw images — (PDF) [file pone.0273746.s001.pdf]

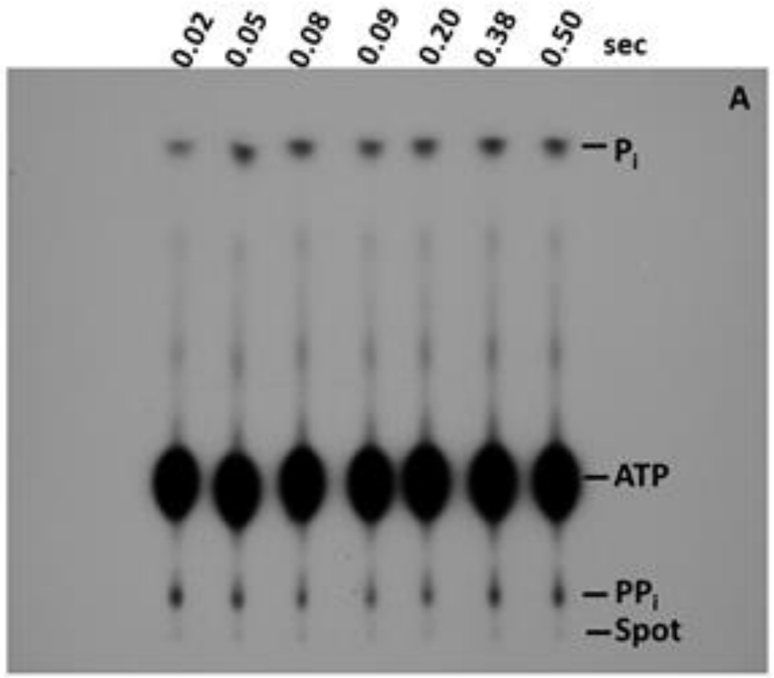

Fig 8. Control for QF-TLC studies illustrating the purity of [γ-^{32}P]ATP and that there is no time-dependent variation in the intensity of the band corresponding to [γ-^{32}P]PP$_i$. **A.** In the autoradiogram, each lane corresponds to a quench at the indicated time. The concentration of [γ-^{32}P]ATP [60 µCi/pmol] after mixing was 50 µM. **From figure 8 in the manuscript.**

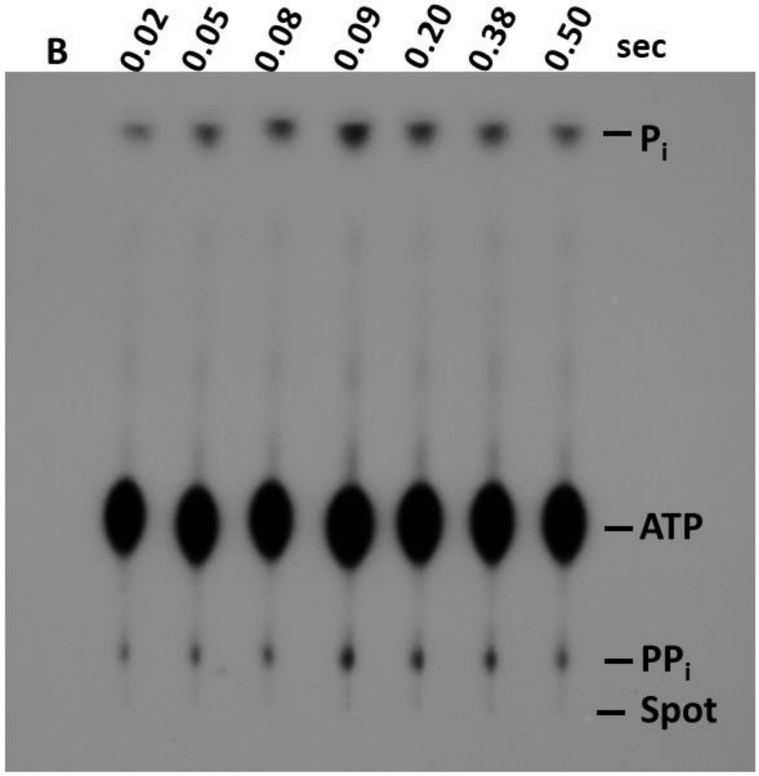

Repeat of experiment given in Fig. 8. Control for QF-TLC studies illustrating the purity of [γ-^{32}P]ATP. In the autoradiogram, each lane corresponds to a quench at the indicated time. The concentration of [γ-^{32}P]ATP [60 µCi/pmol] after mixing was 50 µM at 25°C. Details of the experiment are provided in the main body of the manuscript.

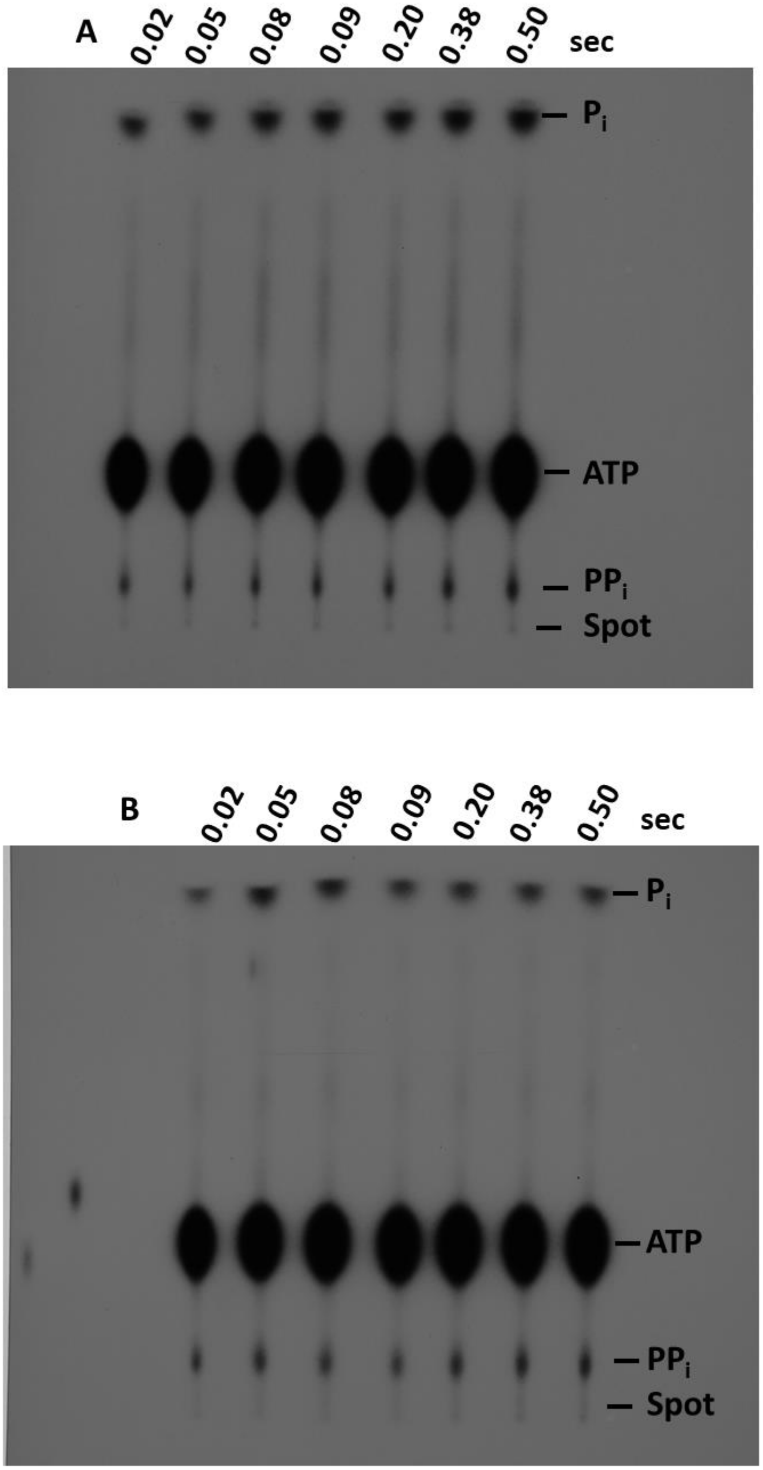

Fig 9. QF-TLC analysis of pyrophosphate release during one and two rounds of the NAC. The autoradiograms correspond to the time-dependent release of $[^{32}P]PP_i$ in the presence of (**A**) 0.5 µM EC and 50 µM $[\gamma\text{-}^{32}P]ATP$ [60 µCi/pmol] after mixing or (**B**) 0.5 µM EC with 50 µM $[\gamma\text{-}^{32}P]ATP$ [60 µCi/pmol] and 50 µM CTP, respectively, after mixing at 25°C. **<u>From figure 9 in the manuscript.</u>**

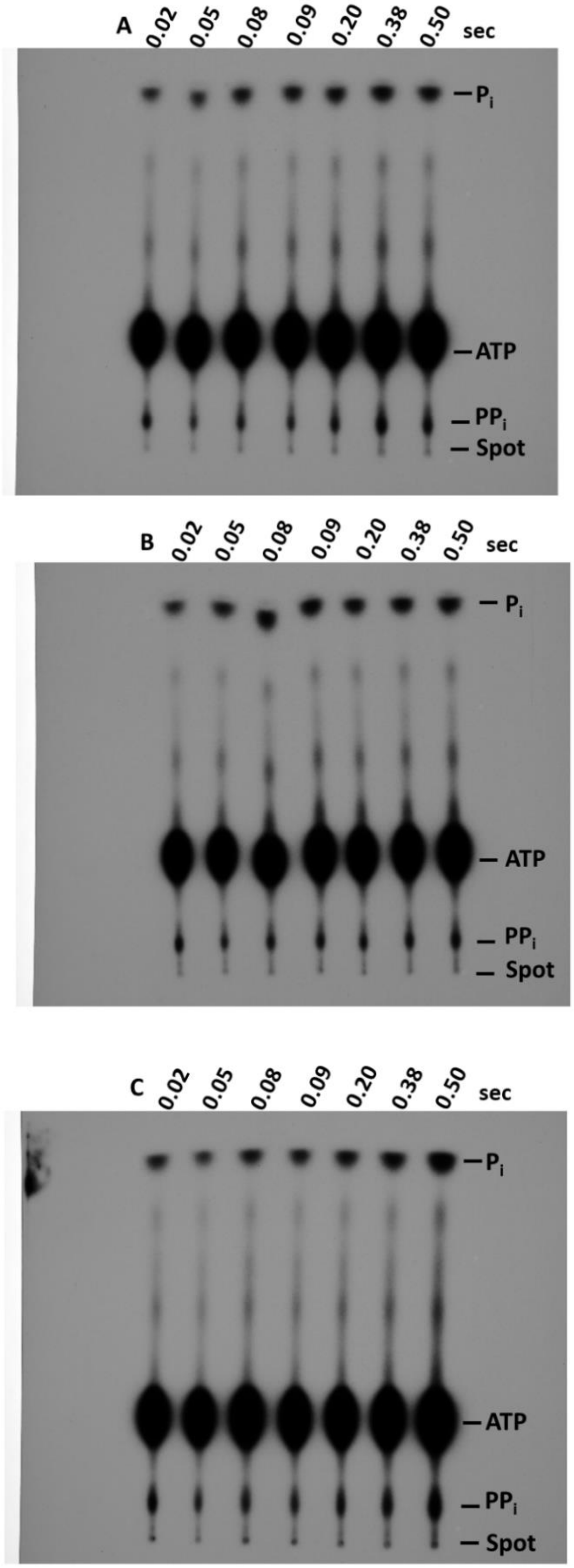

Three repeats of experiment in Fig. 9A. QF-TLC analysis of pyrophosphate release during one round of the NAC. The autoradiograms correspond to the time-dependent release of [^{32}P]PP$_i$ in the presence of 0.5 µM EC and 50 µM [γ-^{32}P]ATP [60 µCi/pmol] after mixing at 25°C. Times are indicated at the top of the autoradiograms. Details of the experiment are provided in the main body of the manuscript.

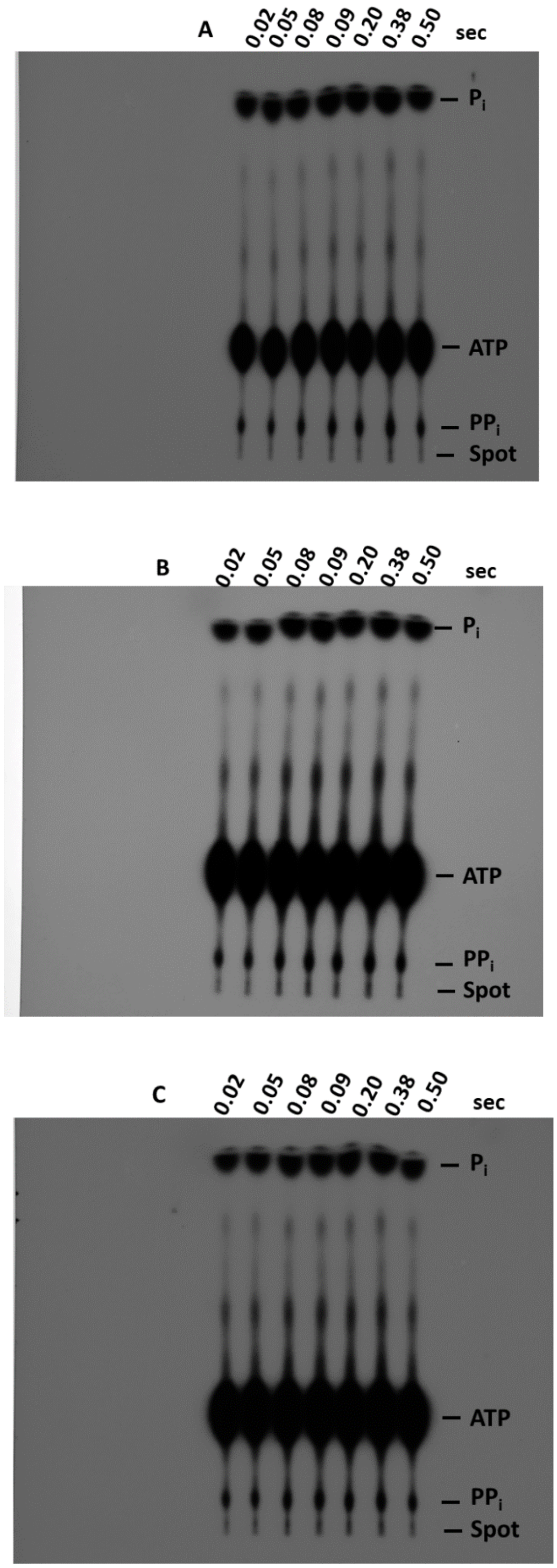

Three repeats of experiment in Fig. 9B. QF-TLC analysis of pyrophosphate release during two rounds of the NAC. The autoradiograms correspond to the time-dependent release of [^{32}P]PP$_i$ in the presence of 0.5 µM EC, 50 µM [γ-^{32}P]ATP [60 µCi/pmol] and 50 µM CTP, respectively, after mixing at 25°C. Times are indicated at the top of the autoradiograms. Details of the experiment are provided in the main body of the manuscript.

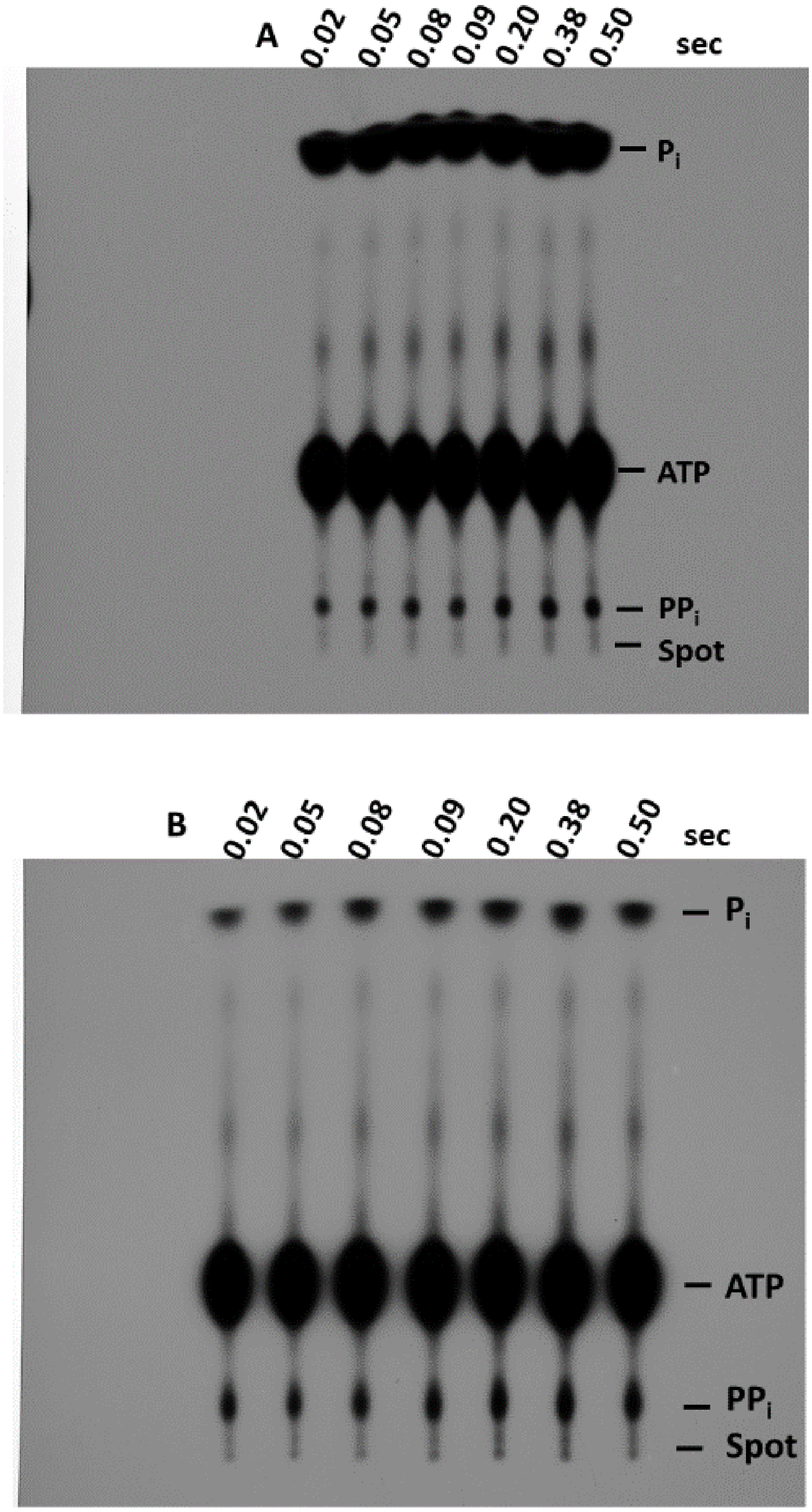

Fig 10. QF-TLC results for pyrophosphate release in the presence of [γ-^{32}P]ATP and UTP (A) as well as [γ-^{32}P]ATP and GTP (B)during one round of the NAC. The autoradiograms correspond to the time-dependent release of [^{32}P]PP_i in the presence of (**A**) 0.5 µM EC, 50 µM [γ-^{32}P]ATP [60 µCi/pmol] and 50 µM UTP after mixing at 25°C and (**B**) 0.5 µM EC with 50 µM [γ-^{32}P]ATP [60 µCi/pmol] and 50 µM GTP, after mixing at 25°C. . **<u>From figure 10 in the manuscript.</u>**

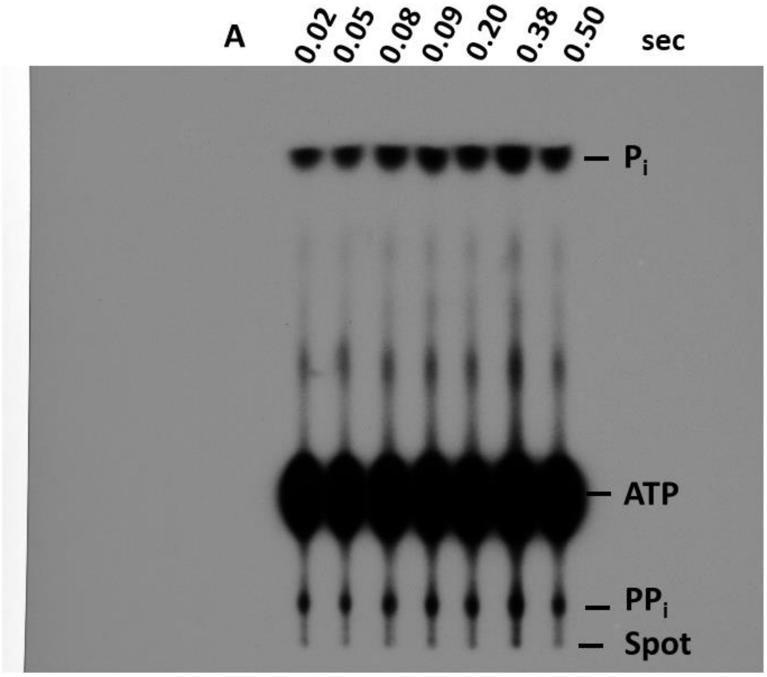

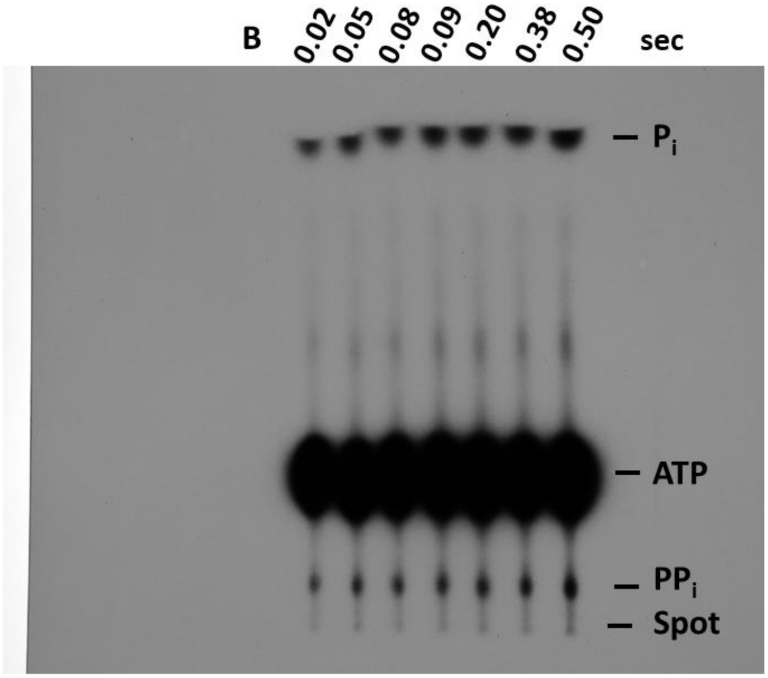

Two repeats of experiment in Fig. 10A. QF-TLC results for pyrophosphate release in the presence of [γ-^{32}P]ATP and UTP) during one round of the NAC. The autoradiograms correspond to the time-dependent release of [^{32}P]PP_i in the presence of 0.5 μM EC, 50 μM [γ-^{32}P]ATP [60 μCi/pmol] and 50 μM UTP after mixing at 25°C. Times are indicated at the top of the autoradiograms. Details of the experiment are provided in the main body of the manuscript.

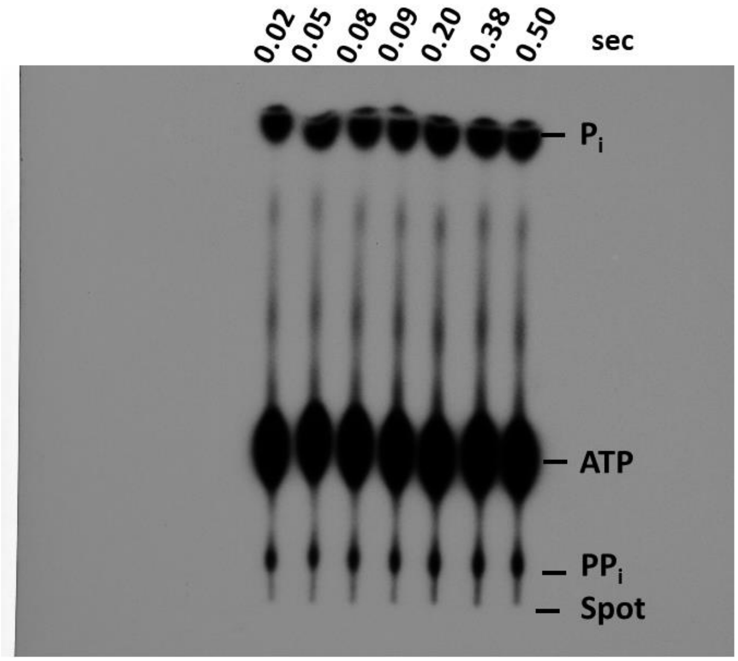

One repeat of experiment in Fig. 10B. QF-TLC results for pyrophosphate release in the presence of [γ-^{32}P]ATP and GTP during one round of the NAC. The autoradiogram corresponds to the time-dependent release of [^{32}P]PP$_i$ in the presence of 0.5 µM EC with 50 µM [γ-^{32}P]ATP [60 µCi/pmol] and 50 µM GTP, after mixing at 25°C. Times are indicated at the top of the autoradiograms. Details of the experiment are provided in the main body of the manuscript.
